# Enhanced Whale Optimization Algorithm with Novel Strategies for 3D TSP Problem

**DOI:** 10.3390/biomimetics10090560

**Published:** 2025-08-22

**Authors:** Yu Zhou, Zijun Hao

**Affiliations:** School of Mathematics and Information Science, North Minzu University, Yinchuan 750021, China; zhouyu20010112@126.com

**Keywords:** improved whale optimization algorithm, a dynamic cluster center-guided search strategy, K-means clustering algorithm, dual-modal population diversity-driven adaptive mutation strategy, pattern search strategy, GPSPositiveBasis2N algorithm

## Abstract

To address the insufficient global search efficiency of the original Whale Optimization Algorithm (WOA), this paper proposes an enhanced variant (ImWOA) integrating three strategies. First, a dynamic cluster center-guided search mechanism based on K-means clustering divides the population into subgroups that conduct targeted searches around dynamically updated centroids, with real-time centroid recalculation enabling evolutionary adaptation. This strategy innovatively combines global optima with local centroids, significantly improving global exploration while reducing redundant searches. Second, a dual-modal diversity-driven adaptive mutation mechanism simultaneously evaluates spatial distribution and fitness-value diversity to comprehensively characterize population heterogeneity. It dynamically adjusts mutation probability based on diversity states, enhancing robustness. Finally, a pattern search strategy (GPSPositiveBasis2N algorithm) is embedded as a periodic optimization module, synergizing WOA’s global exploration with GPSPositiveBasis2N’s local precision to boost solution quality and convergence. Evaluated on the CEC2017 benchmark against the original WOA, eight state-of-the-art metaheuristics, and five advanced WOA variants, ImWOA achieves: (1) optimal mean values for 20/29 functions in 30D tests; (2) optimal mean values for 26/29 functions in 100D tests; and (3) first rank in 3D-TSP validation, demonstrating superior capability for complex optimization.

## 1. Introduction

“Optimization” refers to the process of adjusting decision variables to achieve the optimal value of an objective function under given constraints. As technology advances, modern society faces increasingly complex optimization challenges across multiple domains such as engineering design [1], resource allocation [2], financial risk management [3], data mining [4], material development [5], satellite image analysis [6], energy consumption [7], epidemic control [8], path planning [9], intrusion detection [10], mechanical design [11], feature selection [12,13], machine learning model optimization [14], sustainable applications [15], and resource scheduling [16].

These problems often involve nonlinear, multimodal, high-dimensional, or dynamic scenarios that are difficult to address using traditional methods. This is where metaheuristic algorithms increasingly demonstrate their critical value as core tools for solving complex optimization problems.

The principal advantages of metaheuristic algorithms can be summarized as follows: (1) their applicability to optimization problems lacking analytical objective functions or gradient information; (2) enhanced capability in multi-objective optimization through population-based search that yields multiple solutions in a single execution, contrasting with conventional mathematical programming methods; (3) superior global search capabilities enabled by stochastic exploration; (4) inherent compatibility with parallel computing architectures; and (5) effective handling of mixed-variable optimization problems involving both integer and continuous variables.

Well-known metaheuristic algorithms include: the Barnacles Mating Optimizer (BMO) [17], Earthworm Optimization Algorithm (EOA) [18], Seagull Optimization Algorithm (SOA) [19], Tunicate Swarm Algorithm (TSA) [20], Brain Storm Optimization (BSO) [21], Heap-based optimizer (HBO) [22], Teamwork Optimization Algorithm (TOA) [23], Chaos Game Optimization (CGO) [24], Sine Cosine Algorithm (SCA) [25], Electromagnetic Field Optimization (EFO) [26], Artificial Bee Colony (ABC) [27], Cuckoo Search Algorithm (CSA) [28], Elephant Herding Optimization (EHO) [29], Sea Lion Optimization Algorithm (SLO) [30], and Whale Optimization Algorithm (WOA) [31].

The WOA has gained widespread popularity among researchers due to its strong global search capability, straightforward parameter settings, and rapid convergence rate. The WOA, however, exhibits certain limitations including its susceptibility to local optima entrapment, limited scalability in high-dimensional search spaces, and parameter sensitivity. To address these weaknesses and enhance its optimization capabilities, researchers have conducted extensive modifications through different improvement pathways.

Liang et al. [32] proposed an enhanced WOA (DGSWOA), with core improvements including: population initialization via a Sine–Tent–Cosine mapping to ensure uniform distribution of individuals in the search space; integration of the primary knowledge acquisition phase from the Gaining–Sharing Knowledge (GSK) algorithm to enhance global search capability and avoid local optima; and implementation of a Dynamic Opposition-Based Learning (DOBL) strategy to update population individuals, further increasing solution diversity. These enhancements collectively improve the algorithm’s convergence speed and optimization quality. Liu et al. [33] proposed an improved WOA (DECWOA) with core enhancements including: Sine chaotic mapping for population initialization to enhance diversity; an adaptive inertia weight strategy that dynamically adjusts weights in the position update formula to balance global exploration and local exploitation; and integration of the Differential Evolution (DE) algorithm to improve search speed and accuracy through mutation, crossover, and selection. These innovations effectively address the original algorithm’s tendencies for premature convergence and slow convergence. Chakraborty et al. [34] proposed an improved WOA, whose core enhancements lie in an iterative partitioning strategy that dedicates the first half of iterations to exploration and the latter half to exploitation. During exploration, two modified prey search strategies are introduced to enhance solution diversity. In the exploitation phase, the concept of whale “cooperative hunting” is integrated with original strategies to strengthen local search capability and the convergence rate. Sun et al. [35] proposed an improved WOA (MWOA-CS) with core enhancements including: introducing a nonlinear convergence factor and a cosine function-based inertia weight to dynamically balance the exploration–exploitation capabilities of WOA, and integrating the update mechanism of the Cross Search Optimizer (CSO). During iterations, the algorithm randomly selects each dimension of the optimization problem to execute either the modified WOA or CSO, effectively avoiding local optima while improving convergence speed and precision for large-scale global optimization problems. Shen et al. [36] proposed an improved multi-population evolution-based WOA (MEWOA). Its core innovations include dividing the whale population into three sub-populations—exploration-oriented, exploitation-oriented, and balance-oriented—each employing distinct movement strategies to emphasize global exploration, local exploitation, and exploration–exploitation balance, respectively. By integrating a population evolution strategy that alternates between position updates and evolutionary refinement during iterations, the algorithm effectively enhances population diversity and prevents premature convergence. These advancements significantly strengthen its global search capability and convergence speed in addressing global optimization and engineering design challenges. Li et al. [37] proposed an improved multi-strategy WOA (MWOA), with core enhancements including: introducing an elite opposition-based learning strategy to optimize initial populations, a nonlinear convergence factor to balance exploration and exploitation, Differential Evolution (DE) mutation strategies to strengthen global exploration capability, and a Lévy flight perturbation strategy to enhance search space diversity. These strategies collectively enhance the convergence speed and precision of the original WOA, effectively avoiding local optima while demonstrating stronger competitiveness in tackling complex global optimization problems.

While existing enhancement schemes have improved the performance of the WOA, there remains room for improvement in its global exploration capability and convergence speed. To address these limitations, this study aims to strengthen the WOA algorithm by exploring novel strategies, thereby further advancing its global search capacity, local exploitation capability, and convergence performance. The main contributions and innovations of this work are summarized as follows:

- **A Dynamic Cluster Center-guided Search Strategy Based on the K-means Clustering Algorithm**: This method divides the population into multiple subgroups, with each subgroup conducting searches around its corresponding clustering center. Meanwhile, the clustering centers are recalculated in each iteration to dynamically adjust the centroids, enabling rapid adaptation to population changes and enhancing adaptability and robustness, thus avoiding premature convergence. Additionally, the position update strategy—which integrates both the position of the globally optimal individual and the centroid position of local clusters—demonstrates rapid environmental adaptability. It reduces redundant search operations while improving overall search efficiency, reliably converging to the globally optimal solution.
- **Dual-Modal Population Diversity-Driven Adaptive Mutation Strategy**: When individuals in the population exhibit excessive similarity, the algorithm tends to converge prematurely to a local optimum. Traditional methods primarily assess diversity by merely measuring the distances between individuals within the population, which fails to fully capture the diversity of the population. To more comprehensively depict the heterogeneous characteristics of the population, this strategy considers both spatial distribution diversity and fitness value diversity. Additionally, this strategy develops a dynamic mutation mechanism that adjusts the mutation probability in real time based on the population’s diversity state. When diversity is low, mutations are applied with a higher probability to enhance global exploration capabilities; conversely, when diversity is high, mutations are applied with a lower probability to maintain convergence efficiency. Compared to static parameter configurations, this adaptive adjustment mechanism demonstrates stronger robustness and adaptability to specific problems.
- **Pattern Search Strategy Based on the GPSPositiveBasis2N Algorithm**: WOA struggles to perform refined searches in certain complex spaces. This proposed strategy combines WOA with a pattern search method to leverage the strengths of both approaches, thereby enhancing optimization performance. Specifically, WOA can swiftly locate potential optimal regions within the global search space, while the pattern search strategy conducts refined searches within these regions, thus improving overall efficiency. Consequently, this strategy introduces a hybrid optimization framework that integrates the pattern search method, namely the “GPSPositiveBasis2N algorithm,” as a periodic optimization module within the workflow of the WOA. By combining the complementary advantages of global exploration (achieved by WOA) and local precise optimization (achieved by GPSPositiveBasis2N), this framework enhances the solution quality and convergence efficiency.

The structure of this paper is outlined as follows: Section 2 systematically outlines the core mechanisms and procedural framework of the original WOA. Section 3 elaborates on three innovative strategies of the ImWOA, including design motivations, theoretical significance, and mathematical modeling. Section 4 presents quantitative experimental results of ImWOA and benchmark algorithms on the CEC2017 test suite, with comprehensive analysis of key metrics such as convergence behavior and solution accuracy. Section 5 applies ImWOA to three-dimensional Traveling Salesman Problems (TSPs), validating its engineering practicality through performance comparisons with other WOA variants. Finally, Section 6 summarizes the research contributions and proposes potential directions for future work.

## 2. The Original WOA

The WOA is a novel metaheuristic algorithm proposed by Mirjalili and Lewis [31] in 2016, which simulates the hunting behavior of whale populations in nature to find optimal solutions. It primarily consists of three components: prey encirclement, bubble-net attack, and searching for prey.

### 2.1. Prey Encirclement

When hunting, whales form a circle around their prey and progressively reduce the size of this encirclement, causing the individual whales to converge towards the current best solution (the location of the prey). The specific mathematical equations are as follows:

(1) $$X\left(t+1\right)=X^{*}-A\cdot D_{1}$$

(2) $$D_{1}=\left|C\cdot X^{*}-X\left(t\right)\right|$$

where *t* represents the current iteration number, X(t) denotes the position of the whale at the t-th iteration, X(t+1) represents the position of the whale at the (t + 1)-th iteration, and $X^{*}$ signifies the current optimal position of the whale. *A* and *C* are parameters, and their calculation formulas are as follows:

(3) A=2·a·r−a

(4) C=2·r

(5) $$a=2-\frac{2\cdot t}{MaxIter}$$

where *r* represents a random number between 0 and 1, *a* denotes the convergence coefficient, which decreases from 2 to 0 as iterations increase, and MaxIter denotes the maximum number of iterations.

### 2.2. Bubble-Net Attack

During this phase, whales trap their prey by creating a spiral bubble net and simultaneously swim along a spiral path to approach the target. The specific mathematical formulations are as follows:

(6) $$X\left(t+1\right)=D_{2}\cdot e^{bl}\cdot cos\left(2\pi l\right)+X^{*}$$

(7) $$D_{2}=\left|X^{*}-X\left(t\right)\right|$$

where the constant *b* governs the spiral’s logarithmic shaping, and *l* represents a random number within the interval from −1 to 1.

### 2.3. Searching for Prey

During this phase, whales engage in random walking to expand the search range, and the algorithm randomly selects reference individuals to achieve global exploration. The specific mathematical formulations are as follows:

(8) $$X\left(t+1\right)=X_{rand}\left(t\right)-A\cdot D_{3}$$

(9) $$D_{3}=\left|C\cdot X_{rand}\left(t\right)-X\left(t\right)\right|$$

where $X_{rand}\left(t\right)$ designates the positional coordinates of a stochastically selected whale. The parameters *A* and *C* are computed using the relationships defined in Equations (3) and (4), respectively.

The selection of behavioral strategies in the algorithm is controlled by parameters *A* and *p*, where *p* is a random number between 0 and 1. When p<0.5 and $\left|A\right|\geq 1$, searching for prey is executed; when p<0.5 and $\left|A\right|<1$, the prey encirclement strategy is carried out; when p≥0.5, the bubble-net attacking strategy is implemented.

## 3. Proposed ImWOA

This section presents an enhanced variant of the WOA (ImWOA) to address inherent limitations in the original WOA.

### 3.1. A Dynamic Cluster Center-Guided Search Strategy Based on the K-Means Clustering Algorithm

The k-means clustering algorithm divides the population into multiple subgroups, with each subgroup conducting searches around its corresponding cluster center. This mechanism enhances population diversity through spatial partitioning. By recalculating cluster centers in each iteration, the algorithm achieves dynamic adjustment of centroids, enabling rapid adaptation to population changes. Concurrently, it can promptly adjust search directions according to the current population distribution. This strategic framework allows the algorithm to better regulate search orientations during iterative processes, thereby improving both adaptability and robustness. Specifically, the dynamic centroid updating mechanism establishes an adaptive balance between the exploration and exploitation phases, while the subgroup partitioning structure effectively prevents premature convergence through diversified local searches.

The formula for the number of clusters is as follows:

(10) $$K=\sqrt{N}$$

where *N* represents the number of individuals in the population.

For each iteration, the *k*-means clustering algorithm partitions the population into *K* clusters:

(11) $$S^{*}=\left\{S_{1}^{*},S_{2}^{*}\ldots \ldots S_{K}^{*}\right\}$$

Each of these *K* clusters corresponds to a distinct cluster centroid:

(12) $$C^{*}=\left\{C_{1}^{*},C_{2}^{*}\ldots \ldots C_{K}^{*}\right\}$$

For an individual $X_{i}\left\{i\in 1,2\ldots N\right\}$ assigned to cluster $S_{k}\left\{k\in 1,2\ldots K\right\}$, its corresponding cluster center is denoted as $C_{k}\left\{k\in 1,2\ldots K\right\}$. Let *D* denote the dimensionality of each individual in the population, corresponding to the number of decision variables in the *D*-dimensional search space.

The position update formula based on the dynamic cluster center-guided search strategy is presented as follows:

(13) $$x_{i,j}=BestPosition_{j}+\left(BestPosition_{j}-C_{k,j}\right)\cdot {\left(1.5+rand\right)}^{randn}$$

where $i\in \left\{1,2\ldots N\right\}$, $j\in \left\{1,2\ldots D\right\}$ and $k\in \left\{1,2\ldots K\right\}$. In addition, $BestPosition_{j}$ denotes the *j*-th dimensional component of the current optimal individual in the population, and $C_{k,j}$ represents the *j*-th dimensional component of the cluster center $C_{k}$ associated with individual $X_{i}$. In addition, *rand* represents a random number uniformly distributed in the interval [0, 1], serving to introduce stochastic perturbations that prevent premature convergence, and *randn* denotes a random number sampled from a Gaussian distribution $\left(N\left(0,1\right)\right)$, whose purpose is to enhance local escape capability through Gaussian noise injection.

In summary, this dynamic cluster center-guided search strategy achieves a balance between global exploration and local exploitation by partitioning the population into multiple clusters. The efficient clustering process minimizes computational overhead, while the dynamic centroid adaptation mechanism effectively responds to evolving population distributions across iterative phases. The position update strategy—incorporating both the global best individual’s position and the local cluster centroid’s position—demonstrates rapid environmental adaptability and reduces redundant search operations while enhancing overall search efficiency. Such coordinated mechanisms empower the algorithm to reliably converge to the global optimum when addressing complex optimization problems, particularly those with non-convex or multimodal landscapes.

### 3.2. Dual-Modal Population Diversity-Driven Adaptive Mutation Strategy

In evolutionary optimization algorithms, population diversity plays a crucial role in maintaining the balance between exploration and exploitation. When population individuals exhibit excessive similarity, the algorithm tends to suffer premature convergence to local optima, failing to discover superior solutions. Traditional methods primarily evaluate diversity by measuring the distances between individuals within the population. However, such approaches fail to fully reflect the diversity of the population because they overlook the characteristic of fitness. To address this limitation, this study proposes a comprehensive diversity evaluation framework that simultaneously incorporates both spatial distribution diversity and fitness value diversity, thereby enabling more holistic characterization of population heterogeneity.

Furthermore, traditional mutation mechanisms typically employ fixed probability parameters. Building upon our theoretical analysis, we develop a dynamic mutation mechanism driven by dual-modal diversity monitoring (spatial and fitness modalities). This innovation enables real-time adjustment of mutation probability according to population diversity states: when the diversity is low, mutations are applied with a higher probability to enhance the global exploration capability; conversely, when the diversity is high, mutations are applied with a lower probability to maintain the convergence efficiency. This self-adaptive regulation mechanism demonstrates enhanced robustness and problem-specific adaptability compared to static parameter configurations.

The spatial diversity metric of the population, denoted as $Diversity_{position}$, is computed through the following procedure:

First, each parameter dimension of the population is independently normalized using the min–max scaling method, computed as:

(14) $$x_{i,j}^{norm}=\frac{x_{i,j}-min\left(X_{j}\right)}{max\left(X_{j}\right)-min\left(X_{j}\right)}$$

where $i\in \left\{1,2\ldots N\right\}$ and $j\in \left\{1,2\ldots D\right\}$. In addition, $min(X_{j})$ and $max(X_{j})$, respectively, denote the minimum and maximum values of the *j*-th column in the population matrix *X*.

Second, calculate the standard deviation of each column in the column-normalized population matrix $X^{norm}$:

(15) $$\sigma_{j}=\sqrt{\frac{1}{N}\sum_{i=1}^{N}{\left(x_{i,j}^{norm}-\mu_{j}\right)}^{2}}$$

where $j\in \left\{1,2\ldots D\right\}$, and $\mu_{j}$ represents the mean of the *j*-th column of $X^{norm}$, computed as:

(16) $$\mu_{j}=\frac{1}{N}\sum_{i=1}^{N}x_{i,j}^{norm}$$

Finally, calculate the mean of the standard deviations of all columns:

(17) $$Diversity_{position}=\sigma_{mean}=\frac{1}{D}\sum_{j=1}^{D}\sigma_{j}$$

The fitness diversity metric of the population, denoted as $Diversity_{\mathrm{fitness}}$, is computed through the following procedure:

First, let $fitness_{i}\in R$ denote the fitness value of the *i*-th individual in the population, where $i\in \left\{1,2\ldots N\right\}$. The complete fitness set can be expressed as $Fitness=\left\{fitness_{1},fitness_{2}\ldots fitness_{N}\right\}$.

Second, the fitness values are normalized across the population using min–max scaling to establish a unified measurement scale, computed as:

(18) $$fitness_{i}^{norm}=\frac{fitness_{i}-min\left(Fitness\right)}{max(Fitness)-min(Fitness)}$$

where $i\in \left\{1,2\ldots N\right\}$ and *min*(*Fitness*) and *max*(*Fitness*), respectively, denote the minimum and maximum values within the the complete fitness set *Fitness*.

Finally, the fitness diversity metric $Diversity_{fitness}$ is quantified as the standard deviation of the normalized fitness values across the population, computed as:

(19) $$Diversity_{fitness}=\sigma =\sqrt{\frac{1}{N}\sum_{i=1}^{N}{\left(fitness_{i}^{norm}-\mu \right)}^{2}}$$

where μ denotes the mean normalized fitness value, computed as:

(20) $$\mu =\frac{1}{N}\sum_{i=1}^{N}fitness_{i}^{norm}$$

The dynamic mutation probability threshold $P_{m}^{dynamic}$ is derived through weighted fusion of spatial diversity($Diversity_{position}$) and fitness diversity ($Diversity_{fitness}$) metrics:

(21) $$P_{m}^{dynamic}=w_{1}\cdot Diversity_{position}+w_{2}\cdot Diversity_{fitness}$$

where the weighting coefficients are empirically set as $w_{1}=2$ and $w_{2}=1$, reflecting a 2:1 priority ratio for spatial diversity over fitness diversity.

Given the normalized population position data $X^{norm}$ and fitness values $Fitness^{norm}$, combined with the nature of standard deviation metrics under min–max scaling, we analytically derive the ranges of diversity metrics:

Position Diversity Bound:

(22) $$Diversity_{position}\in \left[0,0.25\right]$$

Fitness Diversity Bound:

(23) $$Diversity_{fitness}\in \left[0,0.5\right]$$

Substituting these bounds into Equation (21), the dynamic mutation probability is theoretically confined to:

(24) $$P_{m}^{dynamic}\in \left[0,1\right]$$

The mutation mechanism is activated stochastically when a uniformly distributed random number $rand∼U\left[0,1\right]$ exceeds the dynamic threshold $P_{m}^{dynamic}$, as formalized by:

(25) $$Mutation\;Trigger\;Condition:rand>P_{m}^{dynamic}$$

Probabilistic Interpretation:

1. Low-Diversity Regime ($P_{m}^{dynamic}\to 0$)
  - Implies reduced population diversity:
    (26) $$Diversity_{position}↓,Diversity_{fitness}↓$$
  - Higher activation probability:
    (27) $$P\left(rand>P_{m}^{dynamic}\right)\approx 1-P_{m}^{dynamic}\to 1$$
  - Promotes intensified exploration through frequent mutations.
2. High-Diversity Regime ($P_{m}^{dynamic}\to 1$)
  - Indicates sufficient population diversity:
    (28) $$Diversity_{position}↑,Diversity_{fitness}↑$$
  - Lower activation probability:
    (29) $$P\left(rand>P_{m}^{dynamic}\right)\approx 1-P_{m}^{dynamic}\to 0$$
  - Prioritizes exploitation by suppressing unnecessary mutations.

If the mutation mechanism is triggered, the mutation formula is as follows:

(30) $$X_{i}=X_{i}+{\left(ub-lb\right)}^{◯randn(D)}⊙rand{\left(D\right)}^{\left[-1,1\right]}$$

where $i\in \left\{1,2\ldots N\right\}$. In addition, *ub* and *lb* denote the upper and lower bounds of the decision variables, respectively, both structured as 1×D row vectors. Moreover, randn(D) is a 1×D vector with elements independently sampled from $N\left(0,1\right)$ (standard normal distribution). ◯ denotes the element-wise exponentiation of vectors *ub-lb* and *randn*(*D*) (raising each element of *ub-lb* to the power of the corresponding element in *randn*(*D*)). Additionally, $rand{\left(D\right)}^{\left[-1,1\right]}$ is a 1×D vector with elements uniformly distributed in $\left[-1,1\right]$, and ⊙ denotes the element-wise multiplication (Hadamard product).

This novel mechanism addresses the limitations of single-indicator approaches through dual integration of spatial diversity (reflecting population distribution breadth) and fitness diversity (indicating solution quality disparity). The mutation probability undergoes autonomous adjustment based on real-time population states: when diversity metrics decline ($P_{m}^{dynamic}↑$), intensified perturbations facilitate escaping local optima; conversely, when the diversity indicator increases ($P_{m}^{dynamic}↓$), redundant searches are reduced to accelerate convergence. This dynamic regulation overcomes the empirical limitations of static mutation probability schemes.

In late-stage optimization phases or high-dimensional search spaces, conventional methods frequently suffer premature convergence due to population homogenization. The integrated mutation mechanism circumvents this pitfall through the targeted perturbation operator (Equation (30)), which systematically generates diversified solutions across the search space. This strategic disturbance reinvigorates global exploration capabilities while significantly mitigating the risk of local optima entrapment.

### 3.3. Pattern Search Strategy Based on the GPSPositiveBasis2N Algorithm

The WOA mimics the hunting behavior of humpback whales, exhibiting strong global search capabilities that enable rapid exploration of the solution space. However, in certain complex spaces, it struggles to perform fine-grained searches. By integrating WOA with a pattern search strategy, the advantages of both approaches can be leveraged to enhance optimization performance. Specifically, WOA can quickly identify potential optimal regions in the global scope, while the pattern search strategy can conduct a refined search within these regions, thereby improving overall efficiency.

The traditional pattern search strategy has deficiencies such as being prone to getting stuck in local optima and having relatively low search efficiency. As an improved variant of pattern search, the GPSPositiveBasis2N algorithm demonstrates core advantages over traditional methods in direction set completeness and robustness. A detailed comparison is outlined below.

1. Direction Set Coverage:

Traditional pattern search uses a minimal positive basis (*N* + 1 directions). For example, in 3D space, it might select [+*x*, +*y*, +*z*] and one diagonal direction, potentially failing to cover all optimization directions.

The GPSPositiveBasis2N algorithm employs 2*N* orthogonal directions (both positive and negative along each coordinate axis, e.g., [+*x*, −*x*, +*y*, −*y*, +*z*, −*z*] in 3D space), which ensures comprehensive directional coverage to avoid stagnation in local optima caused by missing directions, while its symmetrical design reduces sensitivity to initial orientations, thereby enhancing search stability.

2. Adaptability to Non-Smooth/Noisy Functions:

Traditional pattern search may frequently become trapped in pseudo-optimal solutions when handling noisy, discontinuous, or non-smooth objective functions due to incomplete direction sets.

The GPSPositiveBasis2N algorithm’s broader direction coverage ensures that even if some directions fail due to noise, others may still identify improved points. This reflects two key advantages: reduced dependency on function smoothness (making it suitable for black-box models or experimental data in engineering optimization) and strong robustness to maintain effective search performance in noisy environments.

3. Global Exploration Capability:

Traditional pattern search relies on step-size contraction strategies. Poor initial step-size selection may lead to premature convergence to local regions.

The GPSPositiveBasis2N algorithm explores the design space more thoroughly at larger step sizes by covering all positive and negative coordinate directions, offering two key advantages: reduced dependency on initial points to enhance global search capability, and faster escape from local optima during step-size adjustments (e.g., through step enlargement).

4. Convergence Guarantee:

While both traditional pattern search and GPSPositiveBasis2N satisfy the convergence theory of pattern search (converging to local minima for smooth functions), the latter’s 2*N* direction set ensures linear independence of the positive basis, guaranteeing progressive convergence even under non-smooth conditions through step-size contraction.

The GPSPositiveBasis2N algorithm follows the structured pattern search framework to iteratively refine solutions. Algorithm 1 presents the pseudocode for the GPSPositiveBasis2N algorithm.
**Algorithm 1** GPSPositiveBasis2N algorithm**Require:**   Objective function $F_{\mathrm{Obj}}$   Initial point $x_{0}\in R^{n}$   Lower bound LoB, upper bound UpB   Initial step size $\Delta_{0}>0$, tolerance τ>0   Maximum iterations $K_{\max}$**Ensure:** Optimal solution $x^{*}$, optimal value $f^{*}$ 1:$x_{\mathrm{best}}\leftarrow x_{0}$ 2:$f_{\mathrm{best}}\leftarrow F_{\mathrm{Obj}}\left(x_{\mathrm{best}}\right)$ 3:$\Delta \leftarrow \Delta_{0}$ 4:Generate positive basis $D={\left[\begin{array}{cc} I_{n} & -I_{n} \end{array}\right]}^{T}$ 5:**for** $k=1\to K_{\max}$ **do** 6:      improved←false 7:      **for** each direction $d_{i}\in D$ **do** 8:            $x_{\mathrm{cand}}\leftarrow x_{\mathrm{best}}+\Delta d_{i}$ 9:            $x_{\mathrm{cand}}\leftarrow \mathrm{clip}\left(x_{\mathrm{cand}},\mathrm{LoB},\mathrm{UpB}\right)$10:           $f_{\mathrm{cand}}\leftarrow F_{\mathrm{Obj}}\left(x_{\mathrm{cand}}\right)$11:           **if** $f_{\mathrm{cand}}<f_{\mathrm{best}}$ **then**12:               $x_{\mathrm{best}}\leftarrow x_{\mathrm{cand}}$13:               $f_{\mathrm{best}}\leftarrow f_{\mathrm{cand}}$14:               improved←true15:          **end if**16:      **end for**17:      **if** improved **then**18:            Δ←1.5Δ               ▷ Step expansion19:      **else**20:            Δ←0.5Δ             ▷ Step contraction21:      **end if**22:      **if** Δ<τ **then**23:            **break**24:      **end if**25:**end for**26:**return** $x_{\mathrm{best}},f_{\mathrm{best}}$

In this study, the initial step size ${△}_{0}$ is set to 1, the maximum number of iterations $K_{\max}$ is assigned as 100, and the convergence tolerance τ is fixed at $1\times 10^{-6}$. Additionally, the initial point $x_{0}$ is configured as the best position $x_{best}^{\left(t\right)}$, where represents the optimal solution identified by the population-based metaheuristic at iteration *t*. A refinement search is performed using the GPSPositiveBasis2N algorithm every 200 iterations, and the output $x_{best}$ from GPSPositiveBasis2N is assigned as the initial optimal position $x_{bestposition}$ for subsequent optimization iterations.

This strategy proposes a hybrid optimization framework where the GPSPositiveBasis2N algorithm is embedded as a periodic refinement module within the WOA workflow. The core idea leverages the complementary strengths of global exploration (via WOA) and local precision (via GPSPositiveBasis2N) to enhance solution quality and convergence efficiency.

### 3.4. Whole Framework for ImWOA

**Figure 1:**
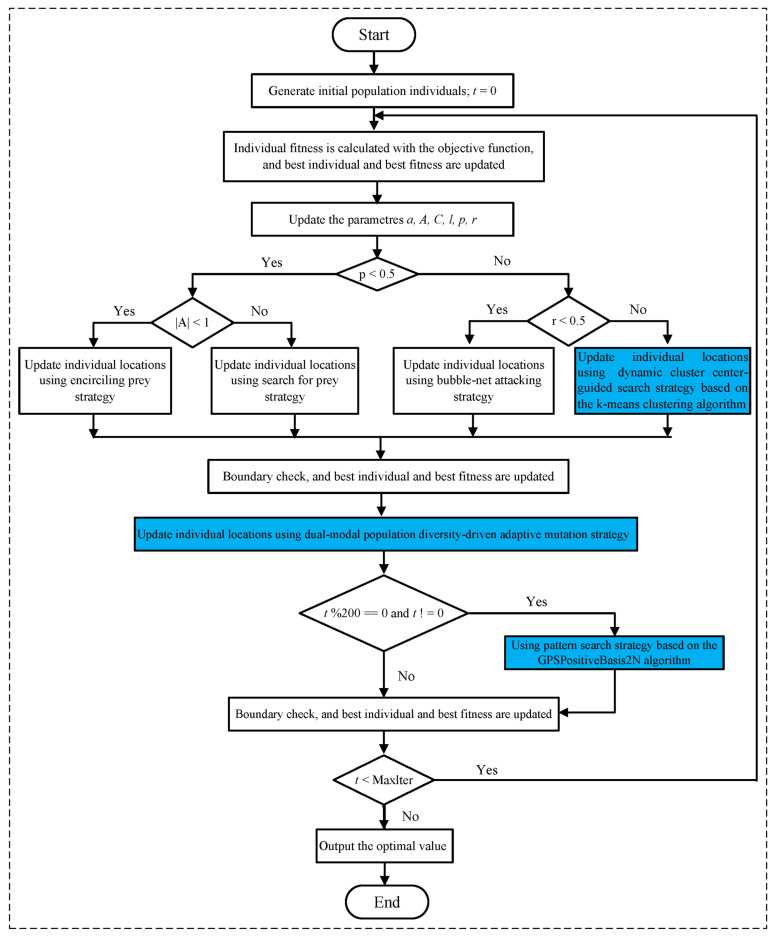
Framework of ImWOA.

The overall structure of the suggested ImWOA approach is depicted in Figure 1.

### 3.5. Computational Complexity Analysis of Algorithms

Time complexity serves as a core metric for evaluating the computational resource demands of an algorithm. Within the context of WOA, we define the whale population size as *N*, the maximum number of iterations as *T*, and the problem dimension as *D*. Consequently, the time complexity of WOA is expressed as O(N∗T∗D). Compared to its predecessor, the ImWOA achieves significant performance enhancements. Through a detailed analysis of its algorithmic refinements—specifically including the dynamic cluster center-guided search strategy based on the K-means clustering algorithm, dual-modal population diversity-driven adaptive mutation strategy, and pattern search strategy based on the GPSPositiveBasis2N algorithm—it becomes evident that these improvements do not necessitate a larger population size, increase the number of iterations, or expand the problem dimension. Therefore, ImWOA maintains the same time complexity of O(N∗T∗D). In summary, ImWOA exhibits identical time complexity to the standard WOA.

### 3.6. Convergence Analysis of ImWOA

The global convergence of ImWOA is guaranteed by the following three theoretical mechanisms, which satisfy the convergence conditions of classical optimization theory:

1. Global convergence guarantee of stochastic search
  According to the Solís–Wets stochastic optimization convergence theorem, ImWOA satisfies two key conditions for probability-1 global convergence:
  - Solution space denseness: Achieved through the diversity-guided mutation mechanism. When population diversity decreases, Gaussian mutation is triggered to ensure solution space coverage.
  - Elitism preservation strategy: The strict historical best solution update mechanism ensures the objective function value is monotonically non-increasing:
    (31) $$f\left({\vec{x}}_{t+1}^{\mathrm{best}}\right)\leq \min\left(f\left({\vec{x}}_{t}^{\mathrm{best}}\right),\min_{i}f\left({\vec{x}}_{i}^{\left(t\right)}\right)\right)$$
2. Convergence inheritance of local search
  The periodically invoked GPSPositiveBasis2N module conforms to the Torczon pattern search convergence framework:
  - Positive basis search direction set $D=\{\pm {\vec{e}}_{i}\}$ generates a dense tangent cone.
  - Step size adaptation rule $\delta_{k+1}=\gamma \delta_{k}$ (γ∈{0.5,1.5}) satisfies $\lim_{k\to \infty }\delta_{k}=0$.
  - A selection strategy that only accepts improved solutions guarantees continuous optimization.
3. Convergence behavior of hybrid architecture
  Based on hybrid optimization theory, the periodic coupling of global exploration (WOA mechanism) and local exploitation (GPS) satisfies:
  (32) $$\lim_{t\to \infty }P(∥{\vec{x}}_{t}-{\vec{x}}^{*}∥<\epsilon )=1,\;\forall \epsilon >0$$
  where global exploration is controlled by an adaptive mutation parameter, and the local search interval $T_{\mathrm{GPS}}=200$ ensures synergistic effects between the two mechanisms.

Note: Given the solution sequence $\left\{{\vec{x}}_{t}\right\}$ generated by ImWOA, the algorithm satisfies:

1. $\forall \epsilon >0,\exists T:P({\vec{x}}_{t}\in R_{\epsilon })>0$ for t>T (Solís–Wets condition).
2. $\lim_{k\to \infty }\inf∥\nabla f\left({\vec{x}}_{k}\right)∥=0$ during GPS phases (Torczon condition).
3. Exponential convergence in probability: $P\left(f\left({\vec{x}}_{t}\right)-f^{*}<\epsilon \right)>1-e^{-\kappa t}$ for t>T(ϵ).

## 4. Experimental Results and Discussions

This study employs the CEC 2017 benchmark function suite [38] to systematically evaluate algorithmic performance across two problem dimensions: 30-dimensional (low-dimensional) and 100-dimensional (high-dimensional) scenarios. The comparative algorithms comprise three categories: (1) the original WOA [31]; (2) eight state-of-the-art metaheuristics: Particle Swarm Optimization (PSO) [39], Biogeography-Based Optimization (BBO) [40], Slime Mould Algorithm (SMA) [41], Differential Evolution (DE) [42], Grey Wolf Optimizer (GWO) [43], Sparrow Search Algorithm (SSA) [44], Harris Hawks Optimization (HHO) [45], and Artificial Bee Colony (ABC) [46]; and (3) five advanced WOA variants: E-WOA [47], IWOA [48], IWOSSA [49], RAV-WOA [50], and WOAAD [51]. All algorithmic parameters strictly adhere to their original literature specifications. Detailed configurations are documented in the respective references to ensure reproducibility.

Experimental settings included a population size of 30 individuals, maximum iterations of 500 generations, and 30 independent runs to eliminate stochastic fluctuations. Algorithms were implemented in Python 3.12 and executed on a computational platform featuring Apple M1 silicon (8-core CPU/7-core GPU) with macOS Sonoma 14.4. The hardware configuration incorporated 8 GB unified memory architecture, while the software stack utilized ARM64 natively compiled scientific computing libraries (NumPy 1.26.0, SciPy 1.11.1), ensuring optimal computational efficiency.

### 4.1. Analysis of Results of CEC2017 Test Functions

The comprehensive statistical results for the 30-dimensional (30 dim) and 100-dimensional (100 dim) benchmark functions from the CEC2017 test suite are detailed in Table 1 and Table 2, respectively. These results encompass the minimum (min), mean, and standard deviation (Std) values derived from thirty independent runs of each algorithm. Notably, the optimal mean values for each benchmark function are highlighted in bold font. Furthermore, the “Total” row at the bottom of Table 1 and Table 2 quantifies the frequency with which each algorithm achieved the optimal mean value across all benchmark functions.

Under the 30-dimensional scenario, the proposed ImWOA demonstrates exceptional global optimization capabilities. As detailed in Table 1, ImWOA achieves optimal mean values on 20 out of 29 benchmark functions, accounting for 68.97% of the total test cases. Crucially, for the remaining nine functions where it does not secure the optimal mean, ImWOA still maintains highly competitive performance: ranking 10th on F8, 5th on both F9 and F13, 2nd on F10/F18/F19, 3rd on F21/F23, and 5th on F26. It is particularly noteworthy that in non-optimal cases, ImWOA consistently ranks within the top 10, with more than half (5/9) securing top three positions. These results robustly validate that ImWOA not only effectively escapes local optima traps in complex optimization problems but also exhibits significantly superior solution stability and algorithmic robustness compared to peer algorithms.

In the more challenging 100-dimensional scenario, the ImWOA demonstrates significantly enhanced high-dimensional optimization capabilities. As presented in Table 2, ImWOA achieves optimal mean values on 26 out of 29 benchmark functions, accounting for 89.66% of the test cases—a 20-percentage-point improvement over the 30-dimensional scenario. Crucially, for the remaining three functions without optimal means, the algorithm maintains elite performance: securing second place on F9, fifth on F8, and fourth on F21. A key observation is that all non-optimal rankings are within the top five. These findings conclusively validate that as problem dimensionality increases, ImWOA exhibits substantially strengthened advantages in solution stability, dimensional scalability, and global exploration capacity, with its unique improvement mechanisms effectively mitigating the “curse of dimensionality” on algorithmic performance.

Integrating experimental results from both the 30-dimensional and 100-dimensional scenarios of the CEC2017 benchmark functions, the ImWOA demonstrates progressively enhanced core competencies. In the baseline 30D environment, ImWOA validates its exceptional ability to escape local optima with 68.97% optimal-function coverage (20/29), while maintaining 100% top-10 rankings for non-optimal functions. When scaling to the more challenging 100D setting, the algorithm achieves remarkable performance escalation—the optimal-function ratio surges to 89.66% (26/29), with all three non-optimal functions securing top five rankings (peak position: second). This unique inverse relationship between dimensionality and performance (dimensionality↑ → optimality↑ → ranking-convergence↑) conclusively demonstrates that through its distinctive population coordination mechanism and adaptive search strategies, ImWOA not only effectively mitigates solution degradation in high-dimensional spaces but also transforms the “curse of dimensionality” into a catalyst for algorithmic evolution, thereby delivering a breakthrough approach for complex optimization problems.

### 4.2. Analysis of the Convergence Behavior of the Algorithms

**Figure 2:**
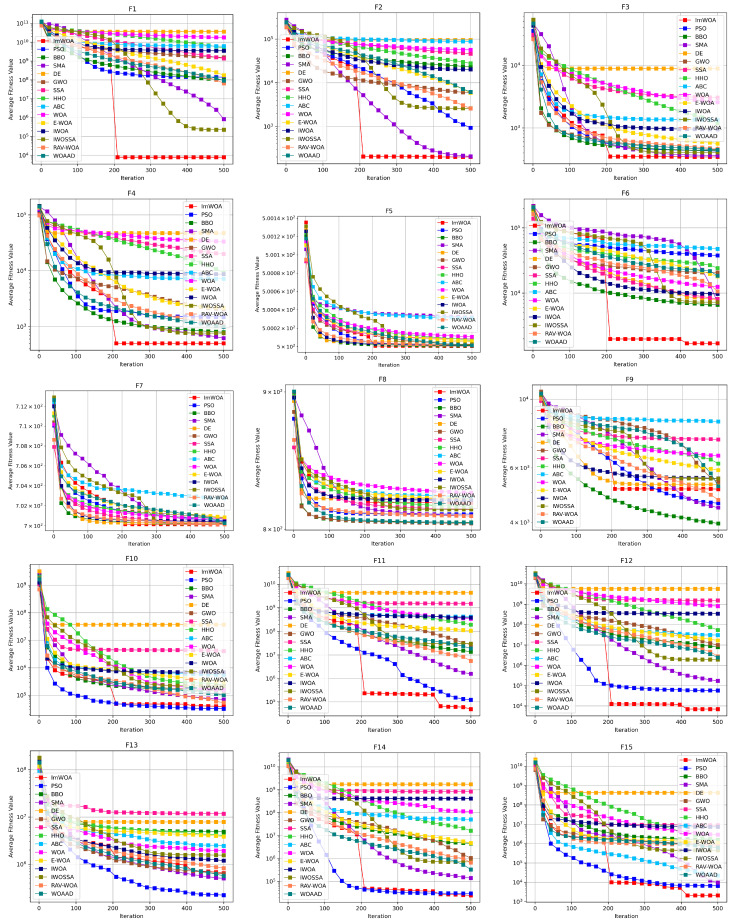

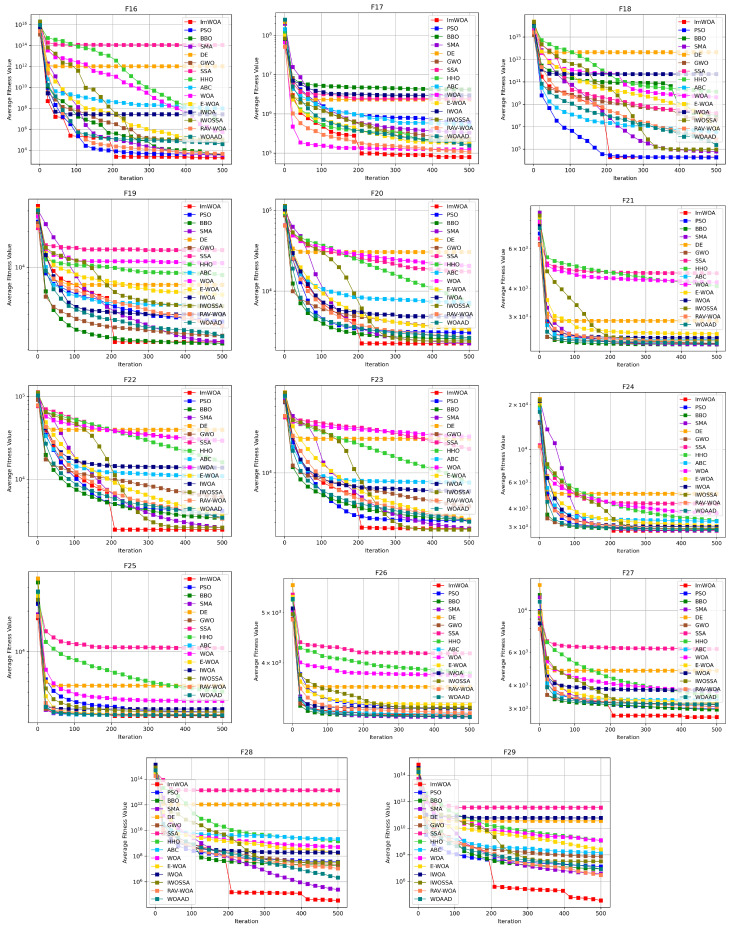
CEC2017 test curves chart (Dim = 30).

**Figure 3:**
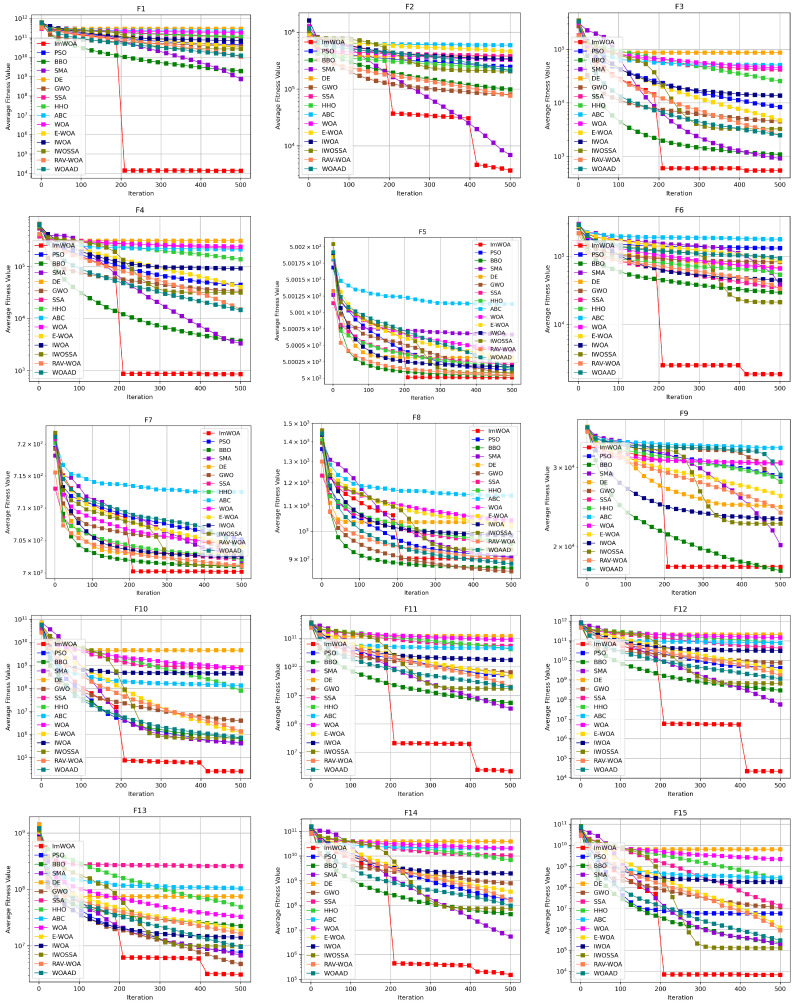

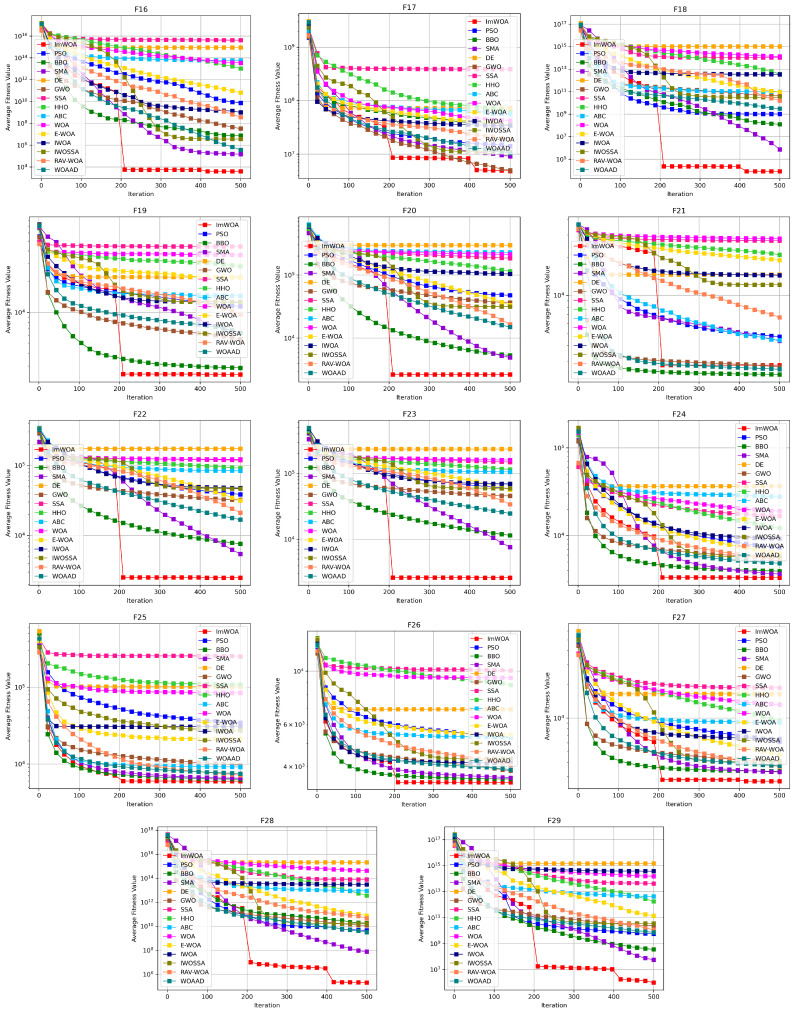
CEC2017 test curves chart (Dim = 100).

To systematically evaluate the comprehensive performance of algorithms in terms of convergence speed and solution efficiency, this study conducts comparative analysis on the convergence characteristics curves of ImWOA versus comparison algorithms under both 30-dimensional and 100-dimensional scenarios (Figure 2 and Figure 3). The abscissa (x-axis) represents the number of iterations, while the ordinate (y-axis) precisely quantifies the mean fitness values obtained through 30 independent experimental trials. These convergence trajectories not only visually reveal the search dynamics within the solution space, but also quantitatively decode fundamental differences in exploration–exploitation balancing mechanisms through critical features such as curve gradients and steady-state plateaus.

Analysis of convergence characteristics in the 30-dimensional scenario (Figure 2) reveals ImWOA’s superior convergence dynamics. The algorithm demonstrates remarkable convergence acceleration on 11 functions (F1, F2, F3, F4, F6, F11, F12, F15, F27, F28, F29), where its convergence trajectory establishes orders-of-magnitude advantages over competitors during early iterations, ultimately locating global optima with enhanced solution quality. Crucially, on 12 functions (F5, F7, F10, F14, F16–F20, F22–F23), while not achieving absolute dominance, ImWOA maintains sustained convergence efficacy—its curves consistently reside in the top performance tier. It should be noted that the constrained search space in low-dimensional environments partially inhibits the full deployment of cooperative search mechanisms, leading to transient attenuation of algorithmic superiority on functions F8, F9, and F21. This phenomenon substantiates the nonlinear coupling between dimensionality scale and strategic effectiveness.

Convergence analysis in the 100-dimensional scenario (Figure 3) reveals ImWOA’s paradigm-shifting dimensional adaptability. The algorithm demonstrates exponential convergence acceleration across 19 benchmark functions (F1–F4, F6, F10–F16, F18–F20, F22–F23, F28–F29), establishing dominant convergence trajectories during initial iterations while maintaining exceptional solution quality through accelerated optimization. Crucially, for seven complex-modal functions (F5, F7, F17, F24–F27), ImWOA exhibits sustained evolutionary refinement properties—systematically outperforming peer algorithms in convergence speed while consistently securing superior final solutions. This comprehensive evidence substantiates ImWOA’s unprecedented solution discovery prowess in high-dimensional search spaces.

In summary, within 30-dimensional spaces, the algorithm achieves statistically significant superiority on most benchmark functions despite low-dimensional search constraints. Crucially, in 100-dimensional environments, ImWOA activates dimensional gain effects—demonstrating pan-domain convergence supremacy across 26/30 functions (86.7% superiority coverage) while generating exponential acceleration phenomena in 19 functions, thereby achieving inverse resolution of the “curse of dimensionality”. This scalable transcendence capability from low-dimensional robustness to high-dimensional disruptiveness establishes a theoretically rigorous and practically validated framework for ultra-large-scale black-box optimization.

### 4.3. Wilcoxon Rank-Sum Test

Each algorithm was independently executed 30 times, yielding 30 optimal values per method. A Wilcoxon rank-sum test was conducted between ImWOA’s solution set and those of each comparative algorithm. When the *p*-value fell below the 0.05 significance threshold (visually emphasized in bold), we rejected the null hypothesis, indicating statistically significant performance differences. Conversely, *p*-values exceeding 0.05 denoted statistical equivalence. For cases demonstrating significant differences (*p* < 0.05), we further compared median values: ImWOA’s superior performance was marked “+” when its median was lower, while competitors’ advantage was denoted “−”. Cases without significant difference (*p* > 0.05) received “=” markers. Comprehensive statistical results are presented in Table 3 and Table 4.

As evidenced in Table 3 and Table 4, ImWOA demonstrates statistically significant superiority (*p* < 0.05) in 89.4% of comparative cases under 30-dimensional tests, while exhibiting statistical equivalence (*p* > 0.05) in the remainder. Crucially, when dimensionality escalates to 100D, the performance disparity intensifies markedly: ImWOA achieves significant dominance (*p* < 0.05) in 98% of trials, maintaining equivalence (*p* > 0.05) only in rare edge cases. This dimensionality-driven performance evolution reveals that ImWOA’s inherent advantages in solution robustness and global convergence capability are substantially amplified with increasing problem complexity.

### 4.4. Friedman Test

To systematically evaluate performance differentials among swarm intelligence algorithms, this study employs the Friedman non-parametric test to establish a multi-algorithm comparison framework. This statistical approach quantifies comprehensive algorithm performance through mean rank values, overcoming the limitations of single-metric evaluation, particularly suited for performance ranking in high-dimensional complex optimization problems.

**Figure 4:**
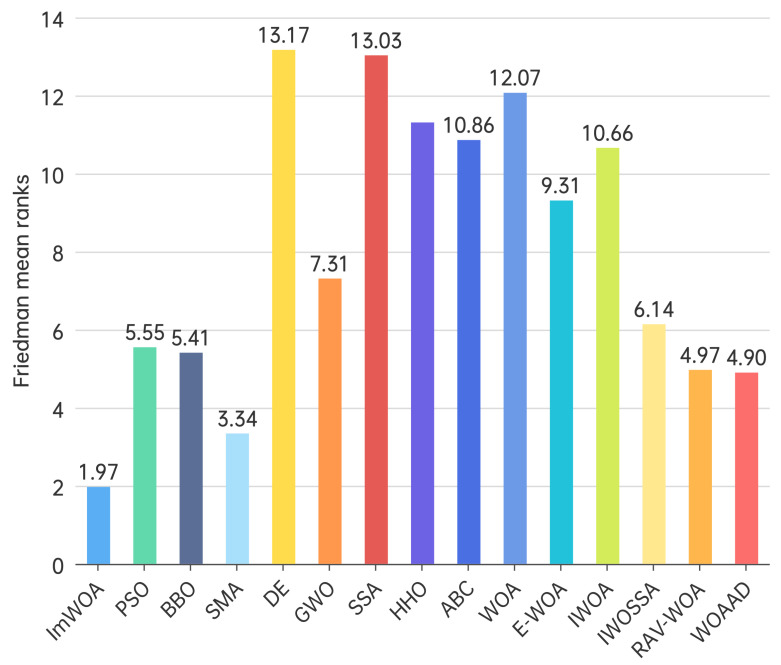
Friedman mean ranks obtained by the employed algorithms (30 dim).

In the 30-dimensional scenario (Figure 4), ImWOA demonstrates commanding dominance with its mean rank value (1.97) substantially lower than all competitors, indicating exceptional optimization stability. Specifically, SMA (3.34), RAV-WOA (4.97), and WOAAD (4.90) form the second echelon within the 3.5–5.0 rank range. Mid-tier performers include BBO (5.41), IWOSSA (6.14), and GWO (7.31). Notably, the classical PSO (5.55) underperforms relative to newer variants. In the lower tier, E-WOA (9.31), ABC (10.86), IWOA (10.66), HHO (11.31), and the baseline WOA (12.07) all exceed the 9.0 threshold, with DE (13.17) and SSA (13.03) occupying the bottom positions. This ranking (*p* < 0.05) rejects the null hypothesis, confirming statistically significant performance differences among algorithms and demonstrating the efficacy of ImWOA’s architectural innovations.

**Figure 5:**
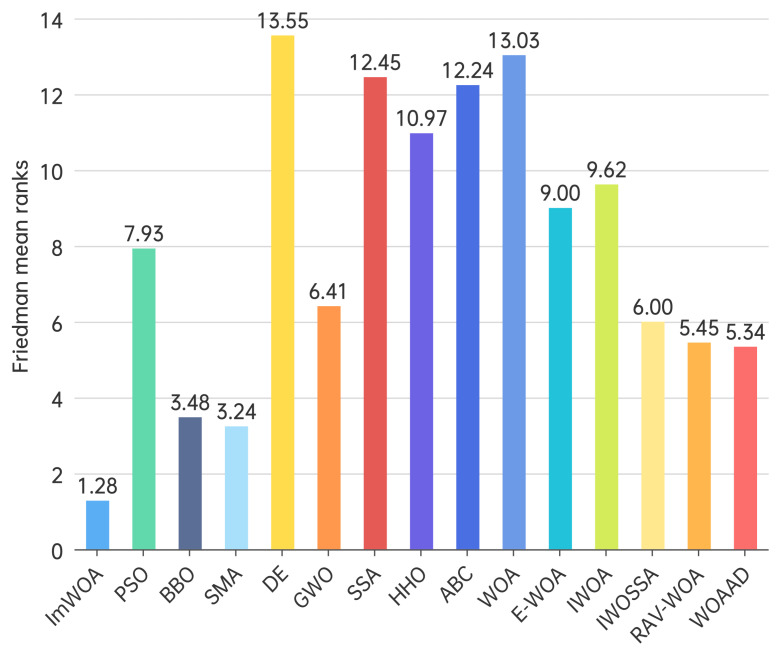
Friedman mean ranks obtained by the employed algorithms (100 dim).

When dimensionality escalates to 100 dimensions, Friedman tests reveal more pronounced algorithmic performance stratification (Figure 5). ImWOA consolidates its dominance with a mean rank value (1.28) reduced by 23% compared to the 30-dimensional scenario, demonstrating exceptional high-dimensional adaptability. Key findings include the following: (1) SMA (3.24) and BBO (3.48) ascend to the secondary tier with over 40% rank improvement; (2) WOAAD (5.34)/RAV-WOA (5.45) maintain a competitive advantage but exhibit narrowed superiority over median performers; (3)classical algorithms show bifurcation: GWO (6.41) demonstrates greater stability than PSO (7.93, rank degradation 43%); and (4) structural reorganization occurs in the lower tier where IWOSSA (6.00) and E-WOA (9.00) significantly outperform their variant IWOA (9.62), while baseline WOA (13.03) and DE (13.55) remain at the bottom. This ranking (*p* < 0.05) confirms that dimensional expansion intensifies algorithmic divergence, highlighting ImWOA’s unique convergence properties in high-dimensional spaces.

### 4.5. Sensitivity of ImWOA to Parameter Variations

Given that the weighting coefficients $w_{1}$ and $w_{2}$ in Equation (21) directly govern the balance between population diversity maintenance and convergence efficiency, sensitivity analysis of these parameters is essential for algorithmic performance evaluation. To this end, this study systematically assesses performance variations under $w_{1}$:$w_{2}$ ratios of {1:1, 2:1, 3:1} using the CEC 2017 benchmark functions in 100-dimensional search spaces. Algorithm ranking follows the standardized average ranking method—calculated by summing ordinal rankings across all benchmark functions and dividing by the total number of functions—where the lowest mean value indicates optimal performance. As evidenced in Table 5, ImWOA achieves a significantly superior average rank at $w_{1}$:$w_{2}$ = 2:1. These results confirm that the 2:1 ratio optimally balances exploration capability and exploitation intensity, whereas lower $w_{1}$ values impair diversity preservation and higher $w_{2}$ values increase premature convergence risks.

## 5. Three-Dimensional TSP

The theoretical depth and practical breadth of the 3D Traveling Salesman Problem (3D-TSP) establish it as a pivotal cross-disciplinary research vehicle. Transcending the dimensional constraints of conventional 2D path planning, it demonstrates unique value in vertical-space dynamic optimization scenarios: urban drone logistics necessitates 3D obstacle avoidance with energy-consumption equilibrium; industrial-scale additive manufacturing relies on spatial trajectory optimization to enhance resource efficiency; autonomous subsea exploration requires efficient visitation of dispersed nodes under oceanic disturbances; and surgical path planning mandates precision and biological-tissue safety. Meanwhile, evolving Urban Air Mobility (UAM) networks demand 3D airspace coordination mechanisms. Addressing these trans-domain challenges, the proposed ImWOA significantly advances global optimization capability and real-time responsiveness in complex environments through innovative 3D solution-space modeling and adaptive computational architecture. This framework provides universal methodological support for strategic fields including intelligent manufacturing, precision medicine, and smart cities, bridging theoretical 3D spatial optimization with engineered applications.

This study implements the ImWOA and multiple comparative algorithms for 3D-TSP resolution, including the original WOA and enhanced variants (E-WOA, IWOA, IWOSSA, RAV-WOA, WOAAD). To mitigate stochastic fluctuations, each algorithm executes 30 independent runs with averaged results serving as the performance benchmark, accompanied by visualization of the optimal path diagram corresponding to the solution closest to the mean value. The city size is set to 100. All urban coordinate data were randomly generated, with three-dimensional coordinates (X, Y, Z) uniformly distributed within the closed interval [100, 5000]. Each dimensional coordinate was independently generated to ensure stochastic spatial distribution and uniform dispersion characteristics.

**Figure 6:**
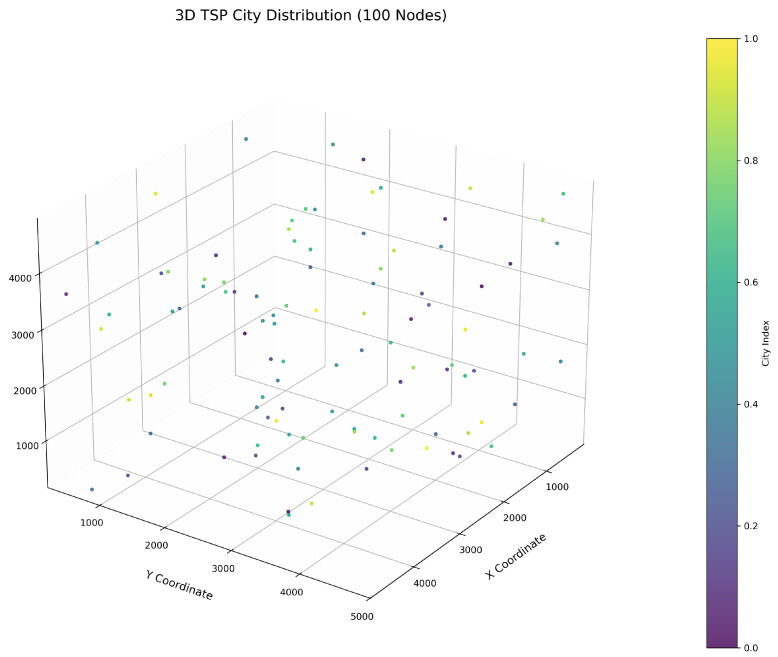
Three-dimensional spatial distribution heatmap of 100 urban nodes.

**Figure 7:**
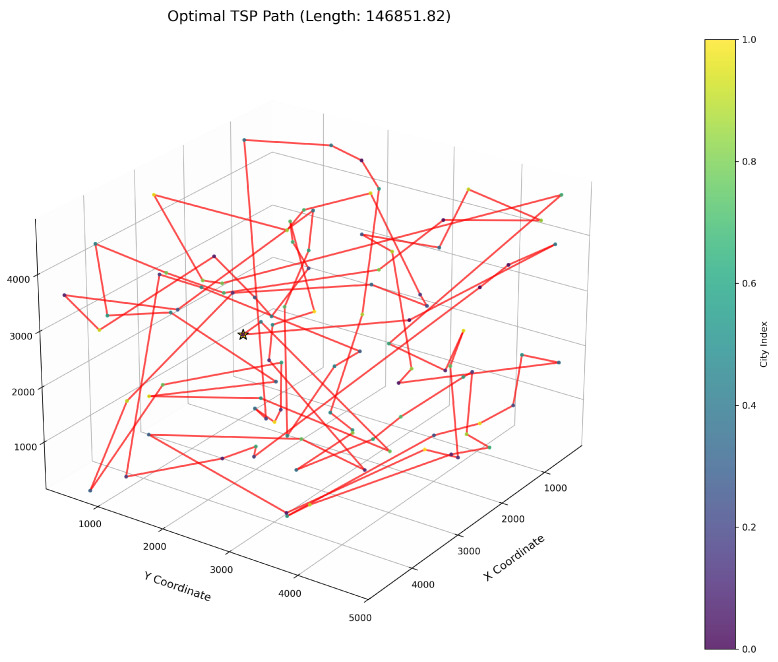
TSP path topology based on average optimal solution (ImWOA).

**Figure 8:**
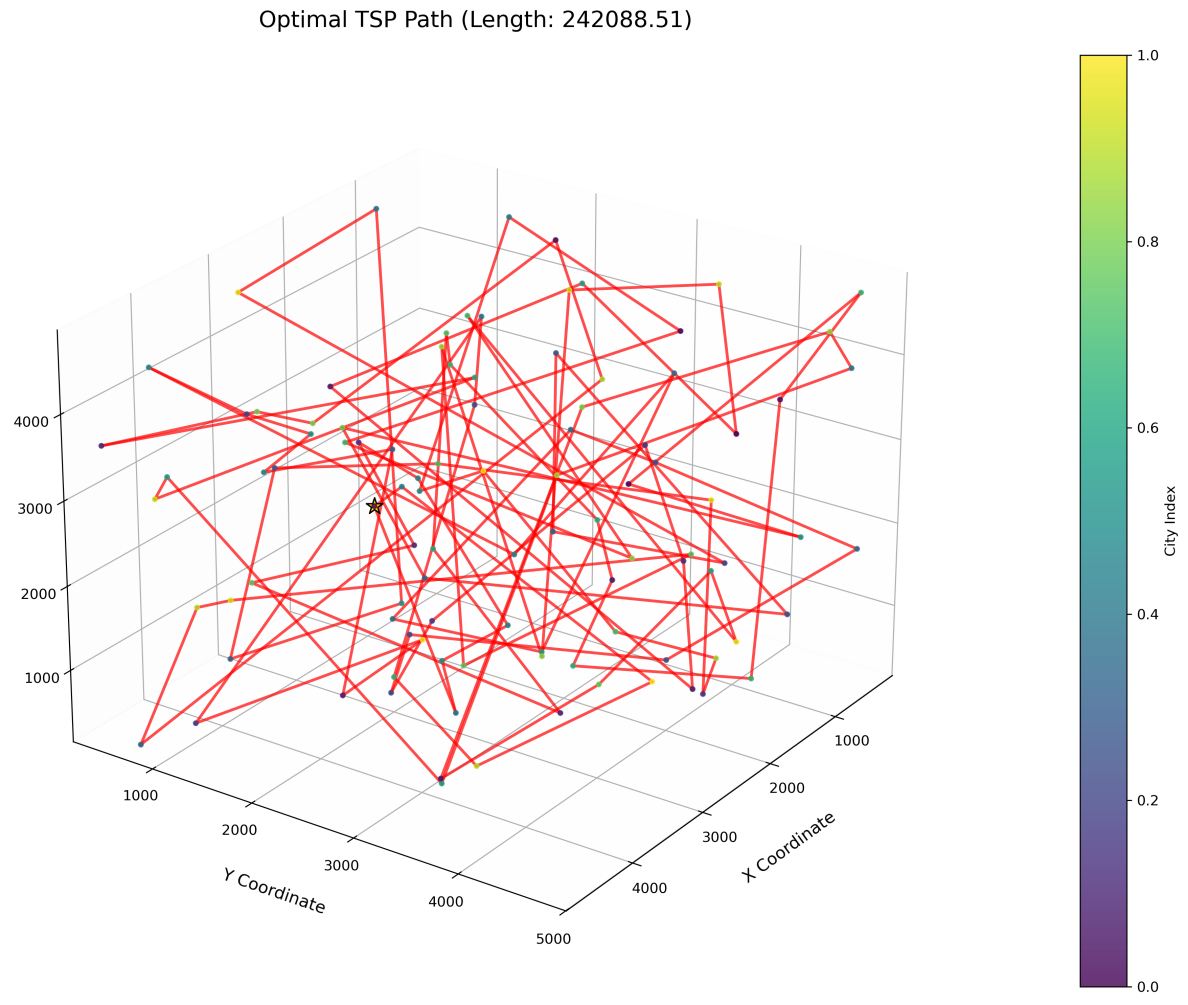
TSP path topology based on average optimal solution (WOA).

**Figure 9:**
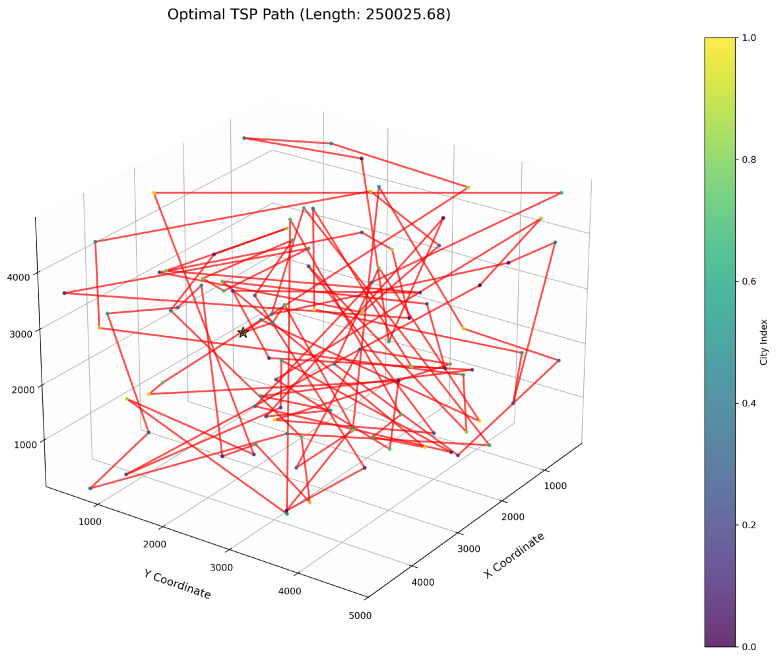
TSP path topology based on average optimal solution (E-WOA).

**Figure 10:**
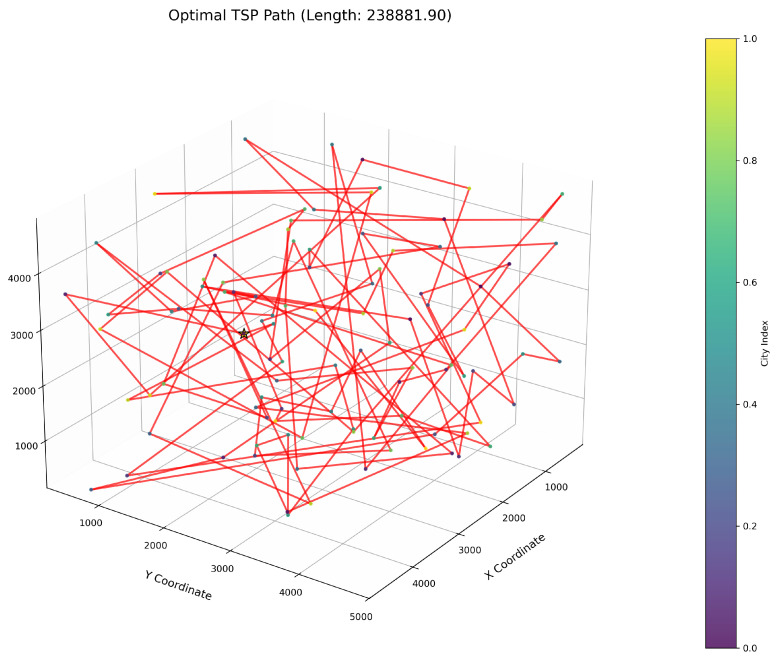
TSP path topology based on average optimal solution (IWOA).

**Figure 11:**
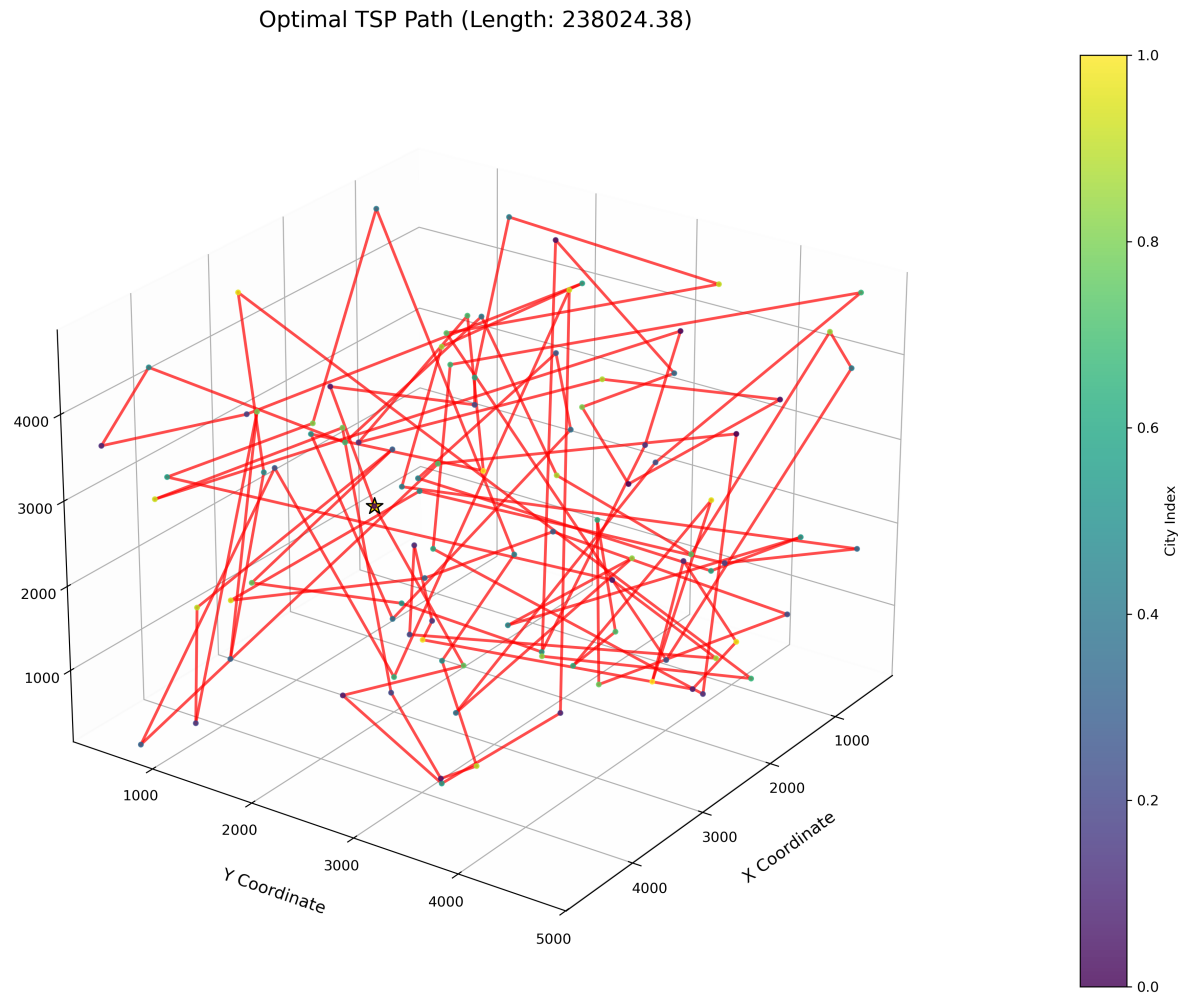
TSP path topology based on average optimal solution (IWOSSA).

**Figure 12:**
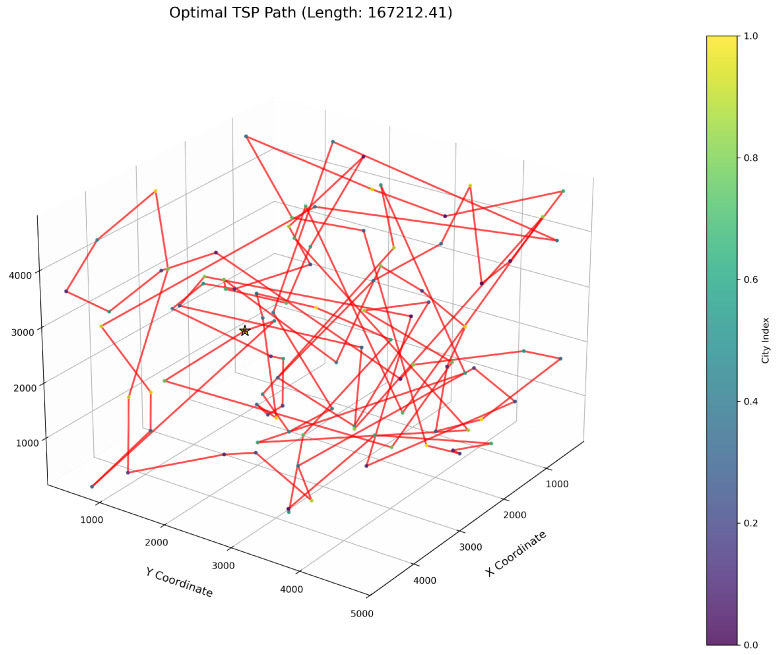
TSP path topology based on average optimal solution (RAV-WOA).

**Figure 13:**
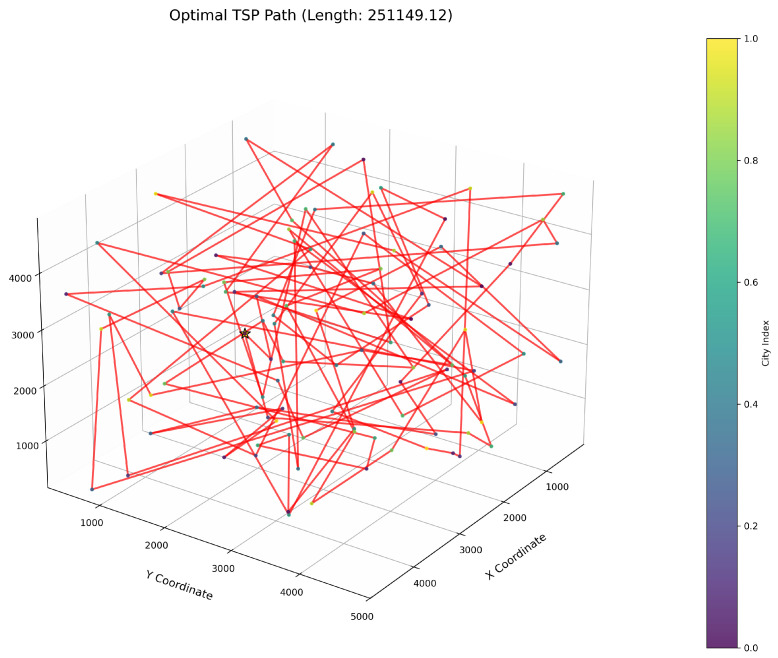
TSP path topology based on average optimal solution (WOAAD).

Figure 6 clearly illustrates the three-dimensional spatial distribution characteristics of 100 urban nodes, Table 6 systematically records the average optimal results of different algorithms from the 30 experiments, and Figure 7, Figure 8, Figure 9, Figure 10, Figure 11, Figure 12 and Figure 13 visually present the actual path planning effect closest to the average optimal solution.

As delineated in Table 6, ImWOA secures top-ranked performance in mean solution quality for three-dimensional Traveling Salesman Problems (TSPs), demonstrating a substantial advantage over benchmark algorithms. Notably, it generates high-precision routing solutions across the test, revealing exceptional exploration capability in complex discrete search spaces. This empirical evidence robustly validates that ImWOA’s unique population cooperation mechanism effectively circumvents local optima traps when handling large-scale combinatorial optimization problems, thereby underscoring its superior potential for engineering applications.

## 6. Conclusions

This study addresses the inherent limitations of the original WOA by proposing an innovative variant, ImWOA, which integrates three synergistic optimization strategies to significantly enhance overall performance:

First Strategy: A dynamic cluster center-guided search mechanism based on K-means clustering. By partitioning the population into multiple subgroups, each subgroup conducts targeted searches around its dynamically updated cluster center. Real-time centroid recalculation during iterations enables dynamic adaptation to population evolution, simultaneously improving adaptability and robustness. This mechanism innovatively integrates global optimum positions with local cluster centroids, effectively suppressing redundant searches while significantly enhancing global exploration efficiency.

Second Strategy: A dual-modal population diversity-driven adaptive mutation mechanism. Transcending traditional single-dimensional distance metrics, this strategy employs dual diversity indicators that simultaneously quantify spatial distribution and fitness value differences, comprehensively characterizing population heterogeneity. The dynamic mutation probability adjustment mechanism, constructed based on real-time diversity states, demonstrates superior environmental adaptability and robustness compared to static parameter configurations.

Third Strategy: A pattern search framework incorporating the GPSPositiveBasis2N algorithm. This hybrid optimization paradigm synergizes WOA’s global exploration capability with pattern search’s local refinement characteristics: WOA rapidly locates potential optimal regions, while the GPSPositiveBasis2N algorithm serves as a periodic optimization module for precision local search. This collaborative “global exploration–local optimization” framework leverages the complementary advantages of both algorithms, achieving breakthrough improvements in solution quality and convergence efficiency.

To comprehensively evaluate the efficacy of the ImWOA, this study employs the CEC2017 benchmark suite for multi-dimensional validation. The experimental design establishes a dual comparison framework: a horizontal comparison with the original WOA and eight state-of-the-art metaheuristic algorithms, alongside a vertical benchmark against five advanced WOA variants. Results demonstrate ImWOA’s exceptional optimization capabilities: in 30-dimensional testing, it achieves optimal mean solutions for 20 out of 29 benchmark functions; when scaling to 100-dimensional problems, it attains leading mean values for 26 of 29 tests. Particularly noteworthy is its first-place ranking in solving the 3D-TSP—a representative combinatorial optimization challenge. These experiments collectively verify that ImWOA not only exhibits superior performance in theoretical benchmarks but also effectively addresses real-world complex optimization problems, highlighting its robust multi-scenario adaptability and broad application potential.

Although ImWOA has demonstrated outstanding optimization performance, its further in-depth research still holds abundant possibilities. Future work will focus on three dimensions as follows:

First, **deepening theoretical mechanisms**, establishing a rigorous mathematical framework to analyze the convergence proof of ImWOA.

Second, **expanding heterogeneous computing paradigms**, exploring integration pathways between ImWOA and cutting-edge technologies such as Graph Neural Networks and quantum computing, thereby constructing a cross-modal optimization framework for high-dimensional complex problems.

Third, **building industrial-grade application ecosystems**, for typical scenarios including dynamic scheduling in smart manufacturing and real-time load allocation in smart grids, developing specialized algorithm engines, and establishing open-source platforms to promote industry–academia–research collaborative innovation.

These explorations will propel ImWOA’s evolution from algorithmic innovation into a universal intelligent optimization infrastructure.

## Figures and Tables

**Table 1:** CEC2017 benchmark results (30 dimensions). Bold indicates optimal values.

Dim = 30																
Func.	Index	ImWOA	PSO	BBO	SMA	DE	GWO	SSA	HHO	ABC	WOA	E-WOA	IWOA	IWOSSA	RAV-WOA	WOAAD
F1	Min	1.81 × 10^2^	1.08 × 10^5^	5.90 × 10^7^	3.52 × 10^5^	1.57 × 10^10^	2.87 × 10^7^	2.85 × 10^8^	2.51 × 10^9^	4.05 × 10^9^	7.62 × 10^9^	7.80 × 10^7^	6.89 × 10^6^	5.13 × 10^3^	2.26 × 10^7^	2.30 × 10^7^
	Mean	**7.90 × 10^3^**	1.40 × 10^8^	9.88 × 10^7^	8.25 × 10^5^	3.65 × 10^10^	1.57 × 10^9^	1.37 × 10^9^	4.89 × 10^9^	6.17 × 10^9^	1.80 × 10^10^	1.75 × 10^8^	3.51 × 10^9^	2.27 × 10^5^	6.54 × 10^7^	9.12 × 10^7^
	Std	7.00 × 10^3^	2.03 × 10^8^	2.98 × 10^7^	3.39 × 10^5^	1.21 × 10^10^	1.37 × 10^9^	2.65 × 10^9^	1.77 × 10^9^	1.38 × 10^9^	7.37 × 10^9^	6.86 × 10^7^	5.21 × 10^9^	3.04 × 10^5^	2.46 × 10^7^	4.69 × 10^7^
	Rank	1	7	6	3	15	10	9	12	13	14	8	11	2	4	5
F2	Min	2.00 × 10^2^	2.00 × 10^2^	8.77 × 10^3^	2.03 × 10^2^	3.60 × 10^4^	7.38 × 10^2^	1.43 × 10^4^	1.60 × 10^4^	6.31 × 10^4^	3.34 × 10^4^	2.81 × 10^3^	2.22 × 10^3^	4.97 × 10^2^	6.97 × 10^2^	1.56 × 10^3^
	Mean	**2.00 × 10^2^**	9.28 × 10^2^	2.31 × 10^4^	2.6 × 10^2^	9.54 × 10^4^	5.94 × 10^3^	4.60 × 10^4^	2.78 × 10^4^	8.81 × 10^4^	5.66 × 10^4^	5.87 × 10^3^	2.02 × 10^4^	2.59 × 10^3^	2.57 × 10^3^	6.14 × 10^3^
	Std	1.50 × 10^−9^	1.49 × 10^3^	9.67 × 10^3^	2.83 × 10^1^	2.53 × 10^4^	3.27 × 10^3^	2.77 × 10^4^	5.78 × 10^3^	1.08 × 10^4^	1.30 × 10^4^	2.32 × 10^3^	1.82 × 10^4^	1.80 × 10^3^	1.27 × 10^3^	2.62 × 10^3^
	Rank	1	3	10	2	15	7	12	11	14	13	6	9	5	4	8
F3	Min	3.03 × 10^2^	3.26 × 10^2^	3.52 × 10^2^	3.32 × 10^2^	2.68 × 10^3^	3.34 × 10^2^	5.00 × 10^2^	7.17 × 10^2^	9.36 × 10^2^	8.80 × 10^2^	3.52 × 10^2^	3.73 × 10^2^	3.30 × 10^2^	3.58 × 10^2^	3.39 × 10^2^
	Mean	**3.45 × 10^2^**	4.29 × 10^2^	4.33 × 10^2^	3.62 × 10^2^	8.80 × 10^3^	4.54 × 10^2^	3.04 × 10^3^	1.20 × 10^3^	1.33 × 10^3^	2.57 × 10^3^	5.63 × 10^2^	9.51 × 10^2^	4.07 × 10^2^	4.62 × 10^2^	4.46 × 10^2^
	Std	2.77 × 10^1^	6.59 × 10^1^	4.86 × 10^1^	3.78 × 10^1^	4.85 × 10^3^	6.69 × 10^1^	4.33 × 10^3^	3.41 × 10^2^	1.57 × 10^2^	1.46 × 10^3^	1.24 × 10^2^	1.12 × 10^3^	4.71 × 10^1^	4.71 × 10^1^	6.50 × 10^1^
	Rank	1	4	5	2	15	7	14	11	12	13	9	10	3	8	6
F4	Min	4.54 × 10^2^	7.37 × 10^2^	6.62 × 10^2^	5.28 × 10^2^	1.87 × 10^4^	6.93 × 10^2^	1.05 × 10^4^	4.30 × 10^3^	5.16 × 10^3^	1.08 × 10^4^	1.19 × 10^3^	1.33 × 10^3^	5.39 × 10^2^	7.85 × 10^2^	8.14 × 10^2^
	Mean	**5.01 × 10^2^**	1.49 × 10^3^	7.93 × 10^2^	6.21 × 10^2^	4.70 × 10^4^	2.02 × 10^3^	2.00 × 10^4^	9.22 × 10^3^	7.13 × 10^3^	3.33 × 10^4^	2.16 × 10^3^	8.68 × 10^3^	7.62 × 10^2^	1.05 × 10^3^	9.92 × 10^2^
	Std	2.91 × 10^1^	6.62 × 10^2^	7.58 × 10^1^	4.70 × 10^1^	1.72 × 10^4^	1.45 × 10^3^	6.00 × 10^3^	2.70 × 10^3^	1.10 × 10^3^	1.00 × 10^4^	3.92 × 10^2^	6.78 × 10^3^	1.12 × 10^2^	1.41 × 10^2^	1.18 × 10^2^
	Rank	1	7	4	2	15	8	13	12	10	14	9	11	3	6	5
F5	Min	5.00 × 10^2^	5.00 × 10^2^	5.00 × 10^2^	5.00 × 10^2^	5.00 × 10^2^	5.00 × 10^2^	5.00 × 10^2^	5.00 × 10^2^	5.00 × 10^2^	5.00 × 10^2^	5.00 × 10^2^	5.00 × 10^2^	5.00 × 10^2^	5.00 × 10^2^	5.00 × 10^2^
	Mean	**5.00 × 10^2^**	5.00 × 10^2^	5.00 × 10^2^	5.00 × 10^2^	5.00 × 10^2^	5.00 × 10^2^	5.00 × 10^2^	5.00 × 10^2^	5.00 × 10^2^	5.00 × 10^2^	5.00 × 10^2^	5.00 × 10^2^	5.00 × 10^2^	5.00 × 10^2^	5.00 × 10^2^
	Std	8.52 × 10^−4^	1.07 × 10^−3^	9.55 × 10^−4^	8.79 × 10^−3^	2.71 × 10^−3^	4.12 × 10^−3^	1.12 × 10^−2^	5.98 × 10^−3^	4.23 × 10^−3^	6.47 × 10^−3^	5.15 × 10^−3^	2.14 × 10^−3^	3.24 × 10^−3^	1.24 × 10^−3^	1.19 × 10^−3^
	Rank	1	3	2	15	9	6	12	11	14	13	10	7	8	5	4
F6	Min	6.01 × 10^2^	2.61 × 10^3^	2.32 × 10^3^	1.49 × 10^3^	1.33 × 10^3^	5.28 × 10^3^	1.00 × 10^3^	6.47 × 10^3^	2.83 × 10^4^	3.54 × 10^3^	3.24 × 10^3^	2.96 × 10^3^	1.18 × 10^3^	2.79 × 10^3^	2.21 × 10^3^
	Mean	**1.71 × 10^3^**	3.76 × 10^4^	6.60 × 10^3^	7.07 × 10^3^	7.79 × 10^3^	2.07 × 10^4^	8.15 × 10^3^	2.41 × 10^4^	4.75 × 10^4^	1.25 × 10^4^	1.80 × 10^4^	9.72 × 10^3^	7.23 × 10^3^	1.06 × 10^4^	1.84 × 10^4^
	Std	1.34 × 10^3^	1.87 × 10^4^	3.02 × 10^3^	4.27 × 10^3^	6.67 × 10^3^	9.36 × 10^3^	4.83 × 10^3^	1.26 × 10^4^	8.49 × 10^3^	7.76 × 10^3^	9.58 × 10^3^	5.16 × 10^3^	5.88 × 10^3^	5.40 × 10^3^	1.03 × 10^4^
	Rank	1	14	2	3	5	12	6	13	15	9	10	7	4	8	11
F7	Min	7.00 × 10^2^	7.00 × 10^2^	7.00 × 10^2^	7.00 × 10^2^	7.00 × 10^2^	7.00 × 10^2^	7.00 × 10^2^	7.00 × 10^2^	7.01 × 10^2^	7.00 × 10^2^	7.00 × 10^2^	7.00 × 10^2^	7.00 × 10^2^	7.00 × 10^2^	7.00 × 10^2^
	Mean	**7.00 × 10^2^**	7.01 × 10^2^	7.00 × 10^2^	7.00 × 10^2^	7.00 × 10^2^	7.01 × 10^2^	7.00 × 10^2^	7.01 × 10^2^	7.03 × 10^2^	7.01 × 10^2^	7.01 × 10^2^	7.00 × 10^2^	7.00 × 10^2^	7.00 × 10^2^	7.00 × 10^2^
	Std	2.74 × 10^−1^	6.81 × 10^−1^	1.23 × 10^−1^	1.37 × 10^−1^	1.73 × 10^−1^	7.91 × 10^−1^	3.88 × 10^−1^	5.00 × 10^−1^	8.15 × 10^−1^	5.03 × 10^−1^	6.00 × 10^−1^	4.40 × 10^−1^	2.00 × 10^−1^	1.34 × 10^−1^	2.17 × 10^−1^
	Rank	1	10	4	3	7	13	8	11	15	12	14	9	5	2	6
F8	Min	8.04 × 10^2^	8.02 × 10^2^	8.01 × 10^2^	8.02 × 10^2^	8.10 × 10^2^	8.01 × 10^2^	8.07 × 10^2^	8.09 × 10^2^	8.13 × 10^2^	8.12 × 10^2^	8.04 × 10^2^	8.08 × 10^2^	8.03 × 10^2^	8.04 × 10^2^	8.01 × 10^2^
	Mean	8.18 × 10^2^	8.10 × 10^2^	8.05 × 10^2^	8.09 × 10^2^	8.21 × 10^2^	**8.04 × 10^2^**	8.14 × 10^2^	8.15 × 10^2^	8.21 × 10^2^	8.25 × 10^2^	8.20 × 10^2^	8.20 × 10^2^	8.14 × 10^2^	8.09 × 10^2^	8.05 × 10^2^
	Std	7.65 × 10^0^	4.33 × 10^0^	3.03 × 10^0^	6.07 × 10^0^	7.15 × 10^0^	2.55 × 10^0^	5.40 × 10^0^	3.43 × 10^0^	3.73 × 10^0^	9.71 × 10^0^	7.53 × 10^0^	7.18 × 10^0^	7.08 × 10^0^	3.84 × 10^0^	3.55 × 10^0^
	Rank	10	6	3	4	14	1	8	9	13	15	12	11	7	5	2
F9	Min	3.62 × 10^3^	2.85 × 10^3^	3.03 × 10^3^	2.45 × 10^3^	4.30 × 10^3^	2.53 × 10^3^	4.41 × 10^3^	4.85 × 10^3^	7.16 × 10^3^	5.09 × 10^3^	4.49 × 10^3^	3.93 × 10^3^	4.13 × 10^3^	3.23 × 10^3^	4.03 × 10^3^
	Mean	5.12 × 10^3^	4.61 × 10^3^	**3.97 × 10^3^**	4.46 × 10^3^	5.31 × 10^3^	5.41 × 10^3^	7.41 × 10^3^	6.19 × 10^3^	8.47 × 10^3^	6.57 × 10^3^	5.90 × 10^3^	5.55 × 10^3^	5.54 × 10^3^	4.73 × 10^3^	5.24 × 10^3^
	Std	7.45 × 10^2^	1.35 × 10^3^	4.37 × 10^2^	6.95 × 10^2^	4.78 × 10^2^	1.92 × 10^3^	1.44 × 10^3^	9.51 × 10^2^	3.59 × 10^2^	1.07 × 10^3^	7.88 × 10^2^	9.94 × 10^2^	8.71 × 10^2^	9.30 × 10^2^	6.92 × 10^2^
	Rank	5	3	1	2	7	8	14	12	15	13	11	10	9	4	6
F10	Min	3.44 × 10^3^	2.68 × 10^3^	1.52 × 10^4^	2.32 × 10^4^	1.59 × 10^5^	7.80 × 10^4^	7.56 × 10^4^	7.79 × 10^4^	2.99 × 10^5^	5.45 × 10^4^	1.40 × 10^5^	4.28 × 10^4^	1.02 × 10^5^	1.02 × 10^4^	5.77 × 10^4^
	Mean	3.91 × 10^4^	**3.22 × 10^4^**	1.43 × 10^5^	7.08 × 10^4^	3.65 × 10^7^	1.44 × 10^5^	4.05 × 10^6^	2.39 × 10^5^	5.52 × 10^5^	1.59 × 10^5^	3.01 × 10^5^	6.86 × 10^5^	2.17 × 10^5^	5.36 × 10^4^	1.01 × 10^5^
	Std	3.01 × 10^4^	1.85 × 10^4^	2.64 × 10^5^	3.38 × 10^4^	8.65 × 10^7^	4.76 × 10^4^	1.18 × 10^7^	2.27 × 10^5^	1.08 × 10^4^	7.37 × 10^4^	1.88 × 10^5^	1.62 × 10^6^	7.12 × 10^4^	3.19 × 10^4^	2.88 × 10^4^
	Rank	2	1	6	4	15	7	14	10	14	8	11	13	9	3	5
F11	Min	7.72 × 10^3^	7.53 × 10^3^	4.13 × 10^6^	5.30 × 10^4^	1.24 × 10^9^	3.71 × 10^6^	1.60 × 10^6^	8.89 × 10^6^	2.82 × 10^8^	1.13 × 10^7^	8.85 × 10^6^	1.75 × 10^5^	1.77 × 10^6^	1.03 × 10^6^	1.93 × 10^6^
	Mean	**4.91 × 10^4^**	1.22 × 10^5^	1.37 × 10^7^	1.57 × 10^6^	4.35 × 10^9^	2.75 × 10^7^	1.50 × 10^9^	1.89 × 10^8^	4.15 × 10^8^	3.39 × 10^8^	1.04 × 10^8^	3.84 × 10^8^	3.03 × 10^7^	5.55 × 10^6^	1.87 × 10^7^
	Std	2.51 × 10^4^	2.01 × 10^5^	8.00 × 10^6^	1.36 × 10^6^	2.18 × 10^9^	1.67 × 10^7^	2.89 × 10^9^	1.31 × 10^8^	7.75 × 10^7^	5.00 × 10^8^	6.43 × 10^7^	8.47 × 10^8^	3.12 × 10^7^	3.77 × 10^6^	1.31 × 10^7^
	Rank	1	2	5	3	15	7	14	10	13	11	9	12	8	4	6
F12	Min	1.31 × 10^3^	2.10 × 10^3^	1.38 × 10^6^	4.43 × 10^4^	4.26 × 10^8^	6.91 × 10^5^	1.08 × 10^5^	1.77 × 10^7^	5.83 × 10^6^	4.13 × 10^7^	1.58 × 10^6^	3.09 × 10^4^	5.92 × 10^5^	3.42 × 10^5^	6.02 × 10^5^
	Mean	**6.95 × 10^3^**	5.75 × 10^4^	7.86 × 10^6^	1.71 × 10^5^	5.65 × 10^9^	1.00 × 10^7^	1.53 × 10^9^	5.36 × 10^7^	3.06 × 10^7^	8.05 × 10^8^	1.66 × 10^7^	3.42 × 10^8^	1.91 × 10^6^	2.73 × 10^6^	2.42 × 10^6^
	Std	5.63 × 10^3^	1.10 × 10^5^	5.13 × 10^6^	1.31 × 10^5^	4.35 × 10^9^	1.94 × 10^7^	3.75 × 10^9^	3.06 × 10^7^	1.68 × 10^7^	8.45 × 10^8^	6.96 × 10^6^	7.43 × 10^8^	8.41 × 10^5^	1.74 × 10^6^	2.06 × 10^6^
	Rank	1	2	7	3	15	8	14	11	10	13	9	12	4	6	5
F13	Min	3.12 × 10^3^	1.86 × 10^4^	1.03 × 10^5^	4.28 × 10^4^	7.00 × 10^5^	1.21 × 10^5^	7.05 × 10^5^	7.84 × 10^5^	5.52 × 10^5^	2.29 × 10^5^	3.04 × 10^5^	1.89 × 10^5^	1.82 × 10^5^	3.03 × 10^4^	2.17 × 10^5^
	Mean	6.45 × 10^5^	**2.22 × 10^5^**	4.85 × 10^6^	5.00 × 10^5^	7.84 × 10^6^	6.24 × 10^5^	1.17 × 10^7^	4.02 × 10^6^	2.44 × 10^6^	1.92 × 10^6^	3.85 × 10^6^	1.19 × 10^6^	1.55 × 10^6^	8.26 × 10^5^	5.62 × 10^5^
	Std	6.77 × 10^5^	1.53 × 10^5^	5.04 × 10^6^	5.39 × 10^5^	7.10 × 10^6^	4.76 × 10^5^	1.44 × 10^7^	2.34 × 10^6^	1.21 × 10^6^	2.03 × 10^6^	3.49 × 10^6^	1.15 × 10^6^	1.48 × 10^6^	6.90 × 10^5^	3.21 × 10^5^
	Rank	5	1	13	2	14	4	15	12	10	9	11	7	8	6	3
F14	Min	4.88 × 10^3^	4.37 × 10^3^	5.99 × 10^5^	7.26 × 10^4^	1.27 × 10^8^	1.30 × 10^5^	1.01 × 10^5^	2.93 × 10^6^	1.75 × 10^7^	6.27 × 10^5^	1.53 × 10^6^	6.60 × 10^4^	2.27 × 10^5^	3.53 × 10^4^	1.09 × 10^5^
	Mean	**2.43 × 10^4^**	2.98 × 10^4^	4.54 × 10^6^	1.39 × 10^5^	1.76 × 10^9^	1.05 × 10^6^	8.37 × 10^8^	1.63 × 10^7^	5.09 × 10^7^	1.11 × 10^8^	4.73 × 10^6^	4.15 × 10^8^	7.10 × 10^5^	6.66 × 10^5^	3.29 × 10^5^
	Std	1.39 × 10^4^	1.48 × 10^4^	3.56 × 10^6^	5.12 × 10^4^	1.09 × 10^9^	2.33 × 10^6^	1.34 × 10^9^	1.07 × 10^7^	2.51 × 10^7^	2.31 × 10^8^	1.60 × 10^6^	1.65 × 10^9^	3.41 × 10^5^	1.07 × 10^6^	1.51 × 10^5^
	Rank	1	2	8	3	15	7	14	10	11	12	9	13	6	5	4
F15	Min	1.51 × 10^3^	1.51 × 10^3^	5.77 × 10^3^	1.77 × 10^3^	4.32 × 10^6^	1.48 × 10^4^	4.65 × 10^3^	6.04 × 10^4^	3.61 × 10^3^	3.40 × 10^4^	5.46 × 10^4^	2.86 × 10^3^	5.18 × 10^3^	4.79 × 10^3^	9.21 × 10^3^
	Mean	**2.10 × 10^3^**	6.59 × 10^3^	1.55 × 10^6^	9.60 × 10^3^	4.23 × 10^8^	8.52 × 10^5^	8.86 × 10^6^	1.48 × 10^6^	2.26 × 10^4^	2.90 × 10^6^	1.11 × 10^6^	7.30 × 10^6^	4.47 × 10^5^	7.32 × 10^5^	6.19 × 10^5^
	Std	6.50 × 10^2^	2.16 × 10^4^	1.85 × 10^6^	6.58 × 10^3^	5.72 × 10^8^	9.47 × 10^5^	3.24 × 10^7^	1.94 × 10^6^	2.26 × 10^4^	4.33 × 10^6^	1.14 × 10^6^	2.71 × 10^7^	7.66 × 10^5^	1.00 × 10^6^	7.79 × 10^5^
	Rank	1	2	11	3	15	8	14	10	4	12	9	13	5	7	6
F16	Min	1.65 × 10^3^	2.44 × 10^3^	2.54 × 10^3^	2.07 × 10^3^	4.74 × 10^4^	2.76 × 10^4^	3.34 × 10^4^	5.53 × 10^4^	2.07 × 10^6^	2.76 × 10^4^	3.54 × 10^4^	4.05 × 10^3^	2.29 × 10^4^	2.71 × 10^3^	6.07 × 10^3^
	Mean	**2.08 × 10^3^**	4.29 × 10^3^	3.78 × 10^3^	3.38 × 10^3^	1.05 × 10^12^	6.03 × 10^4^	1.14 × 10^14^	7.39 × 10^5^	9.84 × 10^7^	5.47 × 10^5^	9.22 × 10^4^	2.69 × 10^7^	1.02 × 10^5^	4.48 × 10^3^	3.81 × 10^4^
	Std	3.95 × 10^2^	3.35 × 10^3^	7.61 × 10^2^	4.42 × 10^2^	3.41 × 10^12^	2.10 × 10^4^	5.55 × 10^14^	2.89 × 10^6^	1.22 × 10^8^	1.62 × 10^6^	3.01 × 10^4^	1.42 × 10^8^	4.95 × 10^4^	1.17 × 10^3^	2.25 × 10^4^
	Rank	1	4	3	2	14	7	15	11	13	10	8	12	9	5	6
F17	Min	3.50 × 10^4^	2.55 × 10^4^	8.12 × 10^4^	5.50 × 10^4^	9.10 × 10^4^	5.68 × 10^4^	4.59 × 10^4^	7.47 × 10^4^	9.36 × 10^4^	3.97 × 10^4^	7.11 × 10^4^	3.76 × 10^4^	6.74 × 10^4^	4.89 × 10^4^	7.04 × 10^4^
	Mean	**7.94 × 10^4^**	6.06 × 10^5^	4.19 × 10^6^	3.32 × 10^5^	2.33 × 10^6^	2.31 × 10^5^	2.45 × 10^6^	1.89 × 10^5^	5.02 × 10^5^	1.22 × 10^5^	1.77 × 10^5^	2.96 × 10^6^	2.17 × 10^5^	1.06 × 10^5^	1.55 × 10^5^
	Std	2.63 × 10^4^	1.18 × 10^6^	4.06 × 10^6^	6.84 × 10^5^	4.23 × 10^6^	3.33 × 10^5^	5.84 × 10^6^	2.22 × 10^5^	2.19 × 10^5^	7.59 × 10^4^	1.42 × 10^5^	1.51 × 10^7^	9.71 × 10^4^	4.89 × 10^4^	6.92 × 10^4^
	Rank	1	11	15	9	12	8	13	6	10	3	5	14	7	2	4
F18	Min	1.86 × 10^3^	2.06 × 10^3^	2.29 × 10^6^	8.58 × 10^3^	2.68 × 10^10^	3.39 × 10^4^	6.40 × 10^4^	4.95 × 10^5^	2.81 × 10^5^	5.28 × 10^5^	1.13 × 10^5^	5.02 × 10^4^	2.58 × 10^4^	3.28 × 10^4^	5.00 × 10^4^
	Mean	1.96 × 10^4^	**1.88 × 10^4^**	1.10 × 10^8^	6.27 × 10^4^	4.39 × 10^13^	4.88 × 10^7^	1.65 × 10^8^	1.32 × 10^10^	3.63 × 10^6^	4.25 × 10^9^	7.23 × 10^6^	4.98 × 10^11^	9.26 × 10^4^	7.41 × 10^5^	2.39 × 10^5^
	Std	2.26 × 10^4^	1.91 × 10^4^	2.00 × 10^8^	2.80 × 10^4^	1.01 × 10^14^	1.44 × 10^8^	5.26 × 10^8^	1.82 × 10^10^	3.67 × 10^6^	8.43 × 10^9^	1.92 × 10^7^	1.71 × 10^12^	3.18 × 10^4^	8.62 × 10^5^	1.94 × 10^5^
	Rank	2	1	10	3	15	9	11	13	7	12	8	14	4	6	5
F19	Min	1.93 × 10^3^	2.10 × 10^3^	2.00 × 10^3^	1.96 × 10^3^	4.10 × 10^3^	2.08 × 10^3^	5.81 × 10^3^	5.16 × 10^3^	3.85 × 10^3^	6.79 × 10^3^	3.17 × 10^3^	2.55 × 10^3^	3.09 × 10^3^	2.44 × 10^3^	2.10 × 10^3^
	Mean	2.33 × 10^3^	3.47 × 10^3^	**2.27 × 10^3^**	2.36 × 10^3^	7.09 × 10^3^	2.64 × 10^3^	1.40 × 10^4^	8.60 × 10^3^	4.69 × 10^3^	1.09 × 10^4^	5.86 × 10^3^	3.97 × 10^3^	4.68 × 10^3^	3.20 × 10^3^	2.60 × 10^3^
	Std	2.82 × 10^2^	1.03 × 10^3^	1.90 × 10^2^	2.62 × 10^2^	2.04 × 10^3^	3.64 × 10^2^	5.38 × 10^3^	1.95 × 10^3^	4.58 × 10^2^	3.17 × 10^3^	1.57 × 10^3^	2.20 × 10^3^	1.12 × 10^3^	5.83 × 10^2^	3.26 × 10^2^
	Rank	2	7	1	3	12	5	15	13	10	14	11	8	9	6	4
F20	Min	2.10 × 10^3^	2.10 × 10^3^	2.27 × 10^3^	2.12 × 10^3^	5.32 × 10^3^	2.47 × 10^3^	3.91 × 10^3^	3.34 × 10^3^	4.98 × 10^3^	4.80 × 10^3^	2.27 × 10^3^	2.86 × 10^3^	2.10 × 10^3^	2.27 × 10^3^	2.31 × 10^3^
	Mean	**2.26 × 10^3^**	3.08 × 10^3^	2.54 × 10^3^	2.27 × 10^3^	3.06 × 10^4^	3.41 × 10^3^	1.75 × 10^4^	6.88 × 10^3^	7.17 × 10^3^	2.06 × 10^4^	3.36 × 10^3^	4.87 × 10^3^	2.42 × 10^3^	2.65 × 10^3^	2.64 × 10^3^
	Std	9.98 × 10^1^	6.18 × 10^2^	7.75 × 10^1^	1.56 × 10^2^	1.07 × 10^4^	8.96 × 10^2^	1.01 × 10^4^	2.09 × 10^3^	9.47 × 10^2^	1.06 × 10^4^	6.71 × 10^2^	2.11 × 10^3^	3.11 × 10^2^	2.68 × 10^2^	2.03 × 10^2^
	Rank	1	7	4	2	15	9	13	11	12	14	8	10	3	6	5
F21	Min	2.20 × 10^3^	2.31 × 10^3^	2.26 × 10^3^	2.26 × 10^3^	2.55 × 10^3^	2.28 × 10^3^	2.67 × 10^3^	2.91 × 10^3^	2.35 × 10^3^	2.74 × 10^3^	2.38 × 10^3^	2.31 × 10^3^	2.29 × 10^3^	2.27 × 10^3^	2.28 × 10^3^
	Mean	2.28 × 10^3^	2.38 × 10^3^	**2.27 × 10^3^**	2.27 × 10^3^	2.88 × 10^3^	2.30 × 10^3^	4.68 × 10^3^	4.10 × 10^3^	2.38 × 10^3^	4.27 × 10^3^	2.52 × 10^3^	2.42 × 10^3^	2.35 × 10^3^	2.29 × 10^3^	2.29 × 10^3^
	Std	4.20 × 10^1^	6.07 × 10^1^	2.86 × 10^0^	4.29 × 10^0^	2.84 × 10^2^	1.43 × 10^1^	1.45 × 10^3^	7.21 × 10^2^	1.25 × 10^1^	1.16 × 10^3^	1.18 × 10^2^	9.06 × 10^1^	4.01 × 10^1^	1.24 × 10^1^	9.60 × 10^0^
	Rank	3	9	1	2	12	6	15	13	8	14	11	10	7	5	4
F22	Min	2.40 × 10^3^	2.42 × 10^3^	2.87 × 10^3^	2.49 × 10^3^	2.16 × 10^4^	3.19 × 10^3^	6.78 × 10^3^	7.18 × 10^3^	9.13 × 10^3^	1.69 × 10^4^	2.94 × 10^3^	3.31 × 10^3^	2.31 × 10^3^	2.98 × 10^3^	2.91 × 10^3^
	Mean	**2.47 × 10^3^**	4.17 × 10^3^	3.40 × 10^3^	2.59 × 10^3^	3.96 × 10^4^	6.14 × 10^3^	2.92 × 10^4^	1.61 × 10^4^	1.09 × 10^4^	2.94 × 10^4^	3.73 × 10^3^	1.38 × 10^4^	2.62 × 10^3^	3.36 × 10^3^	3.45 × 10^3^
	Std	9.63 × 10^1^	1.25 × 10^3^	2.99 × 10^2^	1.11 × 10^2^	9.90 × 10^3^	1.99 × 10^3^	1.39 × 10^4^	6.52 × 10^3^	6.76 × 10^2^	8.22 × 10^3^	5.00 × 10^2^	7.20 × 10^3^	3.43 × 10^2^	2.17 × 10^2^	3.42 × 10^2^
	Rank	1	8	5	2	15	9	13	12	10	14	7	11	3	4	6
F23	Min	2.40 × 10^3^	2.54 × 10^3^	2.93 × 10^3^	2.45 × 10^3^	1.30 × 10^4^	2.75 × 10^3^	4.69 × 10^3^	5.12 × 10^3^	7.17 × 10^3^	1.27 × 10^4^	2.88 × 10^3^	3.36 × 10^3^	2.40 × 10^3^	2.98 × 10^3^	2.87 × 10^3^
	Mean	2.60 × 10^3^	3.17 × 10^3^	3.19 × 10^3^	2.58 × 10^3^	2.28 × 10^4^	4.45 × 10^3^	1.79 × 10^4^	7.88 × 10^3^	7.92 × 10^3^	2.41 × 10^4^	3.21 × 10^3^	6.41 × 10^3^	**2.53 × 10^3^**	3.13 × 10^3^	3.06 × 10^3^
	Std	2.87 × 10^2^	7.32 × 10^2^	1.36 × 10^2^	2.83 × 10^1^	5.28 × 10^3^	1.66 × 10^3^	9.15 × 10^3^	1.89 × 10^3^	4.52 × 10^2^	5.59 × 10^3^	2.70 × 10^2^	2.93 × 10^3^	9.09 × 10^1^	1.06 × 10^2^	1.30 × 10^2^
	Rank	3	6	7	2	14	9	13	11	12	15	8	10	1	5	4
F24	Min	2.82 × 10^3^	2.84 × 10^3^	2.84 × 10^3^	2.82 × 10^3^	3.69 × 10^3^	2.85 × 10^3^	3.04 × 10^3^	3.06 × 10^3^	3.09 × 10^3^	3.22 × 10^3^	2.91 × 10^3^	2.84 × 10^3^	2.84 × 10^3^	2.84 × 10^3^	2.83 × 10^3^
	Mean	**2.83 × 10^3^**	2.88 × 10^3^	2.89 × 10^3^	2.84 × 10^3^	5.02 × 10^3^	2.92 × 10^3^	4.28 × 10^3^	3.30 × 10^3^	3.28 × 10^3^	3.72 × 10^3^	3.04 × 10^3^	3.00 × 10^3^	2.93 × 10^3^	2.88 × 10^3^	2.87 × 10^3^
	Std	9.14 × 10^0^	3.85 × 10^1^	3.37 × 10^1^	2.87 × 10^1^	8.43 × 10^2^	7.88 × 10^1^	1.11 × 10^3^	1.43 × 10^2^	8.57 × 10^1^	3.46 × 10^2^	8.30 × 10^1^	1.86 × 10^2^	4.97 × 10^1^	2.15 × 10^1^	2.80 × 10^1^
	Rank	1	5	6	2	15	7	14	12	11	13	10	9	8	4	3
F25	Min	3.33 × 10^3^	3.33 × 10^3^	3.34 × 10^3^	3.33 × 10^3^	3.94 × 10^3^	3.34 × 10^3^	5.52 × 10^3^	3.81 × 10^3^	3.34 × 10^3^	3.43 × 10^3^	3.34 × 10^3^	3.34 × 10^3^	3.34 × 10^3^	3.39 × 10^3^	3.34 × 10^3^
	Mean	**3.33 × 10^3^**	3.56 × 10^3^	3.35 × 10^3^	3.37 × 10^3^	5.59 × 10^3^	3.40 × 10^3^	1.06 × 10^4^	5.06 × 10^3^	3.37 × 10^3^	4.31 × 10^3^	3.42 × 10^3^	3.74 × 10^3^	3.55 × 10^3^	3.40 × 10^3^	3.37 × 10^3^
	Std	1.35 × 10^−1^	1.50 × 10^2^	1.28 × 10^1^	1.66 × 10^1^	1.01 × 10^3^	5.05 × 10^1^	3.81 × 10^3^	1.22 × 10^3^	1.10 × 10^1^	7.87 × 10^2^	4.78 × 10^1^	3.66 × 10^2^	2.53 × 10^2^	7.71 × 10^0^	1.76 × 10^1^
	Rank	1	10	2	5	14	6	15	13	3	12	8	11	9	7	4
F26	Min	3.11 × 10^3^	3.18 × 10^3^	3.12 × 10^3^	3.11 × 10^3^	3.26 × 10^3^	3.11 × 10^3^	3.54 × 10^3^	3.24 × 10^3^	3.16 × 10^3^	3.24 × 10^3^	3.15 × 10^3^	3.15 × 10^3^	3.13 × 10^3^	3.11 × 10^3^	3.12 × 10^3^
	Mean	3.17 × 10^3^	3.27 × 10^3^	3.15 × 10^3^	**3.14 × 10^3^**	3.60 × 10^3^	3.15 × 10^3^	4.18 × 10^3^	3.82 × 10^3^	3.19 × 10^3^	3.78 × 10^3^	3.32 × 10^3^	3.27 × 10^3^	3.28 × 10^3^	3.20 × 10^3^	3.14 × 10^3^
	Std	3.70 × 10^1^	5.37 × 10^1^	1.53 × 10^1^	1.93 × 10^1^	2.68 × 10^2^	1.76 × 10^1^	4.07 × 10^2^	3.19 × 10^2^	1.40 × 10^1^	3.21 × 10^2^	9.85 × 10^1^	1.34 × 10^2^	9.92 × 10^1^	4.18 × 10^1^	1.60 × 10^1^
	Rank	5	9	4	1	12	3	15	14	6	13	11	8	10	7	2
F27	Min	2.70 × 10^3^	2.84 × 10^3^	2.79 × 10^3^	2.74 × 10^3^	3.69 × 10^3^	3.10 × 10^3^	3.35 × 10^3^	3.31 × 10^3^	3.25 × 10^3^	3.31 × 10^3^	3.16 × 10^3^	3.25 × 10^3^	2.98 × 10^3^	2.80 × 10^3^	2.89 × 10^3^
	Mean	**2.71 × 10^3^**	3.01 × 10^3^	2.96 × 10^3^	3.08 × 10^3^	4.79 × 10^3^	3.17 × 10^3^	6.24 × 10^3^	3.51 × 10^3^	3.33 × 10^3^	3.73 × 10^3^	3.22 × 10^3^	3.76 × 10^3^	3.18 × 10^3^	3.06 × 10^3^	3.15 × 10^3^
	Std	9.01 × 10^0^	1.30 × 10^2^	1.56 × 10^2^	1.57 × 10^2^	8.50 × 10^2^	3.14 × 10^1^	1.68 × 10^3^	2.13 × 10^2^	3.25 × 10^1^	6.14 × 10^2^	3.59 × 10^1^	5.16 × 10^2^	4.27 × 10^1^	1.45 × 10^2^	6.22 × 10^1^
	Rank	1	3	2	5	14	7	15	11	10	12	9	13	8	4	6
F28	Min	6.26 × 10^3^	1.35 × 10^4^	7.23 × 10^4^	1.07 × 10^4^	8.08 × 10^8^	3.59 × 10^4^	2.16 × 10^9^	6.46 × 10^6^	1.80 × 10^8^	6.11 × 10^5^	5.49 × 10^4^	6.06 × 10^4^	3.88 × 10^4^	1.49 × 10^4^	5.91 × 10^4^
	Mean	**3.45 × 10^4^**	3.56 × 10^7^	1.77 × 10^7^	2.46 × 10^5^	1.07 × 10^12^	2.28 × 10^7^	1.36 × 10^13^	1.49 × 10^9^	2.14 × 10^9^	5.11 × 10^8^	1.78 × 10^8^	1.88 × 10^8^	3.26 × 10^7^	1.08 × 10^7^	2.10 × 10^6^
	Std	3.41 × 10^4^	5.62 × 10^7^	2.64 × 10^7^	2.52 × 10^5^	4.32 × 10^12^	3.23 × 10^7^	5.98 × 10^13^	2.70 × 10^9^	1.14 × 10^9^	5.90 × 10^8^	5.20 × 10^8^	3.14 × 10^8^	7.70 × 10^7^	3.14 × 10^7^	4.56 × 10^6^
	Rank	1	8	5	2	14	6	15	12	13	11	9	10	7	4	3
F29	Min	7.62 × 10^3^	1.99 × 10^4^	9.16 × 10^5^	6.14 × 10^4^	1.76 × 10^9^	1.99 × 10^5^	1.53 × 10^6^	1.85 × 10^7^	2.17 × 10^7^	9.81 × 10^7^	1.44 × 10^6^	2.10 × 10^4^	2.12 × 10^5^	1.01 × 10^5^	2.12 × 10^5^
	Mean	**3.62 × 10^4^**	1.29 × 10^7^	8.60 × 10^6^	3.84 × 10^6^	3.56 × 10^10^	7.56 × 10^7^	3.59 × 10^11^	1.15 × 10^9^	1.44 × 10^8^	1.23 × 10^9^	2.51 × 10^8^	5.97 × 10^10^	3.24 × 10^7^	2.97 × 10^6^	8.31 × 10^6^
	Std	3.22 × 10^4^	2.48 × 10^7^	1.86 × 10^7^	7.05 × 10^6^	6.26 × 10^10^	1.64 × 10^8^	1.91 × 10^12^	1.77 × 10^9^	7.59 × 10^7^	1.19 × 10^9^	3.08 × 10^8^	3.09 × 10^11^	4.17 × 10^7^	4.15 × 10^6^	1.24 × 10^7^
	Rank	1	6	5	3	13	8	15	11	9	12	10	14	7	2	4
**Total**		20	3	3	1	0	1	0	0	0	0	0	0	1	0	0

**Table 2:** CEC2017 benchmark results (100 dimensions). Bold indicates optimal values.

Dim = 100																
Func.	Index	ImWOA	PSO	BBO	SMA	DE	GWO	SSA	HHO	ABC	WOA	E-WOA	IWOA	IWOSSA	RAV-WOA	WOAAD
F1	Min	5.95 × 10^2^	1.49 × 10^10^	1.37 × 10^9^	4.97 × 10^8^	1.99 × 10^11^	2.39 × 10^10^	7.33 × 10^10^	8.70 × 10^10^	1.86 × 10^11^	1.53 × 10^11^	1.95 × 10^10^	4.59 × 10^10^	1.61 × 10^10^	7.53 × 10^9^	4.78 × 10^9^
	Mean	**1.37 × 10^4^**	4.45 × 10^10^	1.92 × 10^9^	7.54 × 10^8^	2.95 × 10^11^	3.73 × 10^10^	1.02 × 10^11^	1.11 × 10^11^	2.21 × 10^11^	1.88 × 10^11^	2.82 × 10^10^	7.29 × 10^10^	2.68 × 10^10^	1.05 × 10^10^	1.22 × 10^10^
	Std	1.14 × 10^4^	1.67 × 10^10^	3.74 × 10^8^	1.70 × 10^8^	5.46 × 10^10^	8.97 × 10^9^	1.36 × 10^10^	1.16 × 10^10^	1.53 × 10^10^	1.88 × 10^10^	4.65 × 10^9^	2.11 × 10^10^	6.69 × 10^9^	2.45 × 10^9^	4.03 × 10^9^
	Rank	1	9	3	2	15	8	11	12	14	13	7	10	6	4	5
F2	Min	2.75 × 10^2^	1.65 × 10^5^	5.87 × 10^4^	4.22 × 10^3^	3.02 × 10^5^	5.51 × 10^4^	2.87 × 10^5^	1.76 × 10^5^	5.22 × 10^5^	2.32 × 10^5^	3.37 × 10^5^	2.25 × 10^5^	1.24 × 10^5^	5.19 × 10^4^	1.54 × 10^5^
	Mean	**3.70 × 10^3^**	2.45 × 10^5^	9.88 × 10^4^	6.93 × 10^3^	3.78 × 10^5^	8.05 × 10^4^	3.78 × 10^5^	2.19 × 10^5^	5.93 × 10^5^	3.27 × 10^5^	4.60 × 10^5^	3.40 × 10^5^	2.06 × 10^5^	7.68 × 10^4^	2.10 × 10^5^
	Std	3.67 × 10^3^	4.22 × 10^4^	2.41 × 10^4^	1.65 × 10^3^	5.38 × 10^4^	1.59 × 10^4^	5.37 × 10^4^	1.85 × 10^4^	3.44 × 10^4^	3.92 × 10^4^	5.90 × 10^4^	9.27 × 10^4^	3.87 × 10^4^	1.16 × 10^4^	3.07 × 10^4^
	Rank	1	9	5	2	13	4	12	8	15	10	14	11	6	3	7
F3	Min	4.35 × 10^2^	2.91 × 10^3^	9.10 × 10^2^	7.77 × 10^2^	4.46 × 10^4^	1.92 × 10^3^	1.38 × 10^4^	1.31 × 10^4^	3.92 × 10^4^	2.67 × 10^4^	3.33 × 10^3^	4.90 × 10^3^	2.15 × 10^3^	1.65 × 10^3^	1.70 × 10^3^
	Mean	**5.48 × 10^2^**	8.36 × 10^3^	1.09 × 10^3^	9.20 × 10^2^	8.71 × 10^4^	4.56 × 10^3^	4.22 × 10^4^	2.57 × 10^4^	5.09 × 10^4^	4.51 × 10^4^	4.73 × 10^3^	1.37 × 10^4^	3.26 × 10^3^	2.49 × 10^3^	2.50 × 10^3^
	Std	5.78 × 10^1^	4.01 × 10^3^	9.94 × 10^1^	9.03 × 10^1^	2.29 × 10^4^	1.38 × 10^3^	2.82 × 10^4^	4.37 × 10^3^	6.00 × 10^3^	1.27 × 10^4^	9.21 × 10^2^	5.72 × 10^3^	6.97 × 10^2^	4.70 × 10^2^	3.89 × 10^2^
	Rank	1	9	3	2	15	7	12	11	14	13	8	10	6	4	5
F4	Min	7.16 × 10^2^	2.40 × 10^4^	3.07 × 10^3^	2.72 × 10^3^	1.80 × 10^5^	1.41 × 10^4^	1.53 × 10^5^	1.11 × 10^5^	1.82 × 10^5^	1.73 × 10^5^	2.70 × 10^4^	5.65 × 10^4^	1.65 × 10^4^	1.03 × 10^4^	8.70 × 10^3^
	Mean	**8.62 × 10^2^**	4.49 × 10^4^	3.72 × 10^3^	3.33 × 10^3^	3.16 × 10^5^	3.37 × 10^4^	2.27 × 10^5^	1.39 × 10^5^	2.17 × 10^5^	2.43 × 10^5^	4.06 × 10^4^	9.28 × 10^4^	3.19 × 10^4^	1.50 × 10^4^	1.47 × 10^4^
	Std	5.91 × 10^1^	1.62 × 10^4^	3.57 × 10^2^	3.77 × 10^2^	5.37 × 10^4^	9.55 × 10^3^	4.40 × 10^4^	1.36 × 10^4^	1.26 × 10^4^	2.69 × 10^4^	6.09 × 10^3^	2.54 × 10^4^	7.31 × 10^3^	3.00 × 10^3^	3.30 × 10^3^
	Rank	1	9	3	2	15	7	13	11	12	14	8	10	6	5	4
F5	Min	5.00 × 10^2^	5.00 × 10^2^	5.00 × 10^2^	5.00 × 10^2^	5.00 × 10^2^	5.00 × 10^2^	5.00 × 10^2^	5.00 × 10^2^	5.00 × 10^2^	5.00 × 10^2^	5.00 × 10^2^	5.00 × 10^2^	5.00 × 10^2^	5.00 × 10^2^	5.00 × 10^2^
	Mean	**5.00 × 10^2^**	5.00 × 10^2^	5.00 × 10^2^	5.00 × 10^2^	5.00 × 10^2^	5.00 × 10^2^	5.00 × 10^2^	5.00 × 10^2^	5.00 × 10^2^	5.00 × 10^2^	5.00 × 10^2^	5.00 × 10^2^	5.00 × 10^2^	5.00 × 10^2^	5.00 × 10^2^
	Std	1.10 × 10^−3^	1.58 × 10^−2^	1.60 × 10^−3^	7.66 × 10^−3^	8.27 × 10^−3^	1.56 × 10^−2^	1.66 × 10^−2^	6.06 × 10^−3^	9.15 × 10^−3^	9.28 × 10^−3^	1.18 × 10^−2^	7.48 × 10^−3^	5.24 × 10^−3^	1.51 × 10^−3^	8.59 × 10^−3^
	Rank	1	6	2	14	12	4	10	8	15	13	11	9	5	3	7
F6	Min	7.72 × 10^2^	3.94 × 10^4^	1.49 × 10^4^	2.15 × 10^4^	2.53 × 10^4^	4.85 × 10^4^	2.02 × 10^4^	2.15 × 10^4^	1.62 × 10^5^	2.24 × 10^4^	5.19 × 10^4^	2.35 × 10^4^	9.58 × 10^3^	1.45 × 10^4^	4.58 × 10^4^
	Mean	**1.88 × 10^3^**	1.32 × 10^5^	2.96 × 10^4^	3.81 × 10^4^	6.78 × 10^4^	8.28 × 10^4^	3.47 × 10^4^	5.38 × 10^4^	1.79 × 10^5^	6.64 × 10^4^	8.53 × 10^4^	4.46 × 10^4^	2.12 × 10^4^	3.33 × 10^4^	9.39 × 10^4^
	Std	9.55 × 10^2^	2.80 × 10^4^	8.38 × 10^3^	1.32 × 10^4^	2.37 × 10^4^	2.03 × 10^4^	1.13 × 10^4^	1.24 × 10^4^	9.28 × 10^3^	1.81 × 10^4^	2.46 × 10^4^	1.28 × 10^4^	9.01 × 10^3^	1.07 × 10^4^	2.84 × 10^4^
	Rank	1	14	3	6	10	11	5	8	15	9	12	7	2	4	13
F7	Min	7.00 × 10^2^	7.01 × 10^2^	7.01 × 10^2^	7.01 × 10^2^	7.02 × 10^2^	7.00 × 10^2^	7.01 × 10^2^	7.01 × 10^2^	7.11 × 10^2^	7.02 × 10^2^	7.02 × 10^2^	7.01 × 10^2^	7.01 × 10^2^	7.01 × 10^2^	7.01 × 10^2^
	Mean	**7.00 × 10^2^**	7.05 × 10^2^	7.01 × 10^2^	7.02 × 10^2^	7.03 × 10^2^	7.03 × 10^2^	7.02 × 10^2^	7.02 × 10^2^	7.13 × 10^2^	7.04 × 10^2^	7.04 × 10^2^	7.02 × 10^2^	7.01 × 10^2^	7.01 × 10^2^	7.03 × 10^2^
	Std	1.73 × 10^−1^	2.76 × 10^0^	3.42 × 10^−1^	8.00 × 10^−1^	7.01 × 10^−1^	2.47 × 10^0^	1.13 × 10^0^	7.02 × 10^−1^	8.93 × 10^−1^	1.21 × 10^0^	1.19 × 10^0^	6.87 × 10^−1^	5.05 × 10^−1^	4.63 × 10^−1^	1.06 × 10^0^
	Rank	1	14	2	5	10	9	7	6	15	13	12	8	4	3	11
F8	Min	8.72 × 10^2^	8.69 × 10^2^	8.22 × 10^2^	8.60 × 10^2^	9.61 × 10^2^	8.28 × 10^2^	9.17 × 10^2^	9.20 × 10^2^	1.09 × 10^3^	9.60 × 10^2^	9.24 × 10^2^	9.31 × 10^2^	8.83 × 10^2^	8.81 × 10^2^	8.43 × 10^2^
	Mean	9.01 × 10^2^	9.07 × 10^2^	8.70 × 10^2^	9.05 × 10^2^	1.03 × 10^3^	**8.60 × 10^2^**	9.57 × 10^2^	9.53 × 10^2^	1.14 × 10^3^	1.04 × 10^3^	1.02 × 10^3^	9.84 × 10^2^	9.34 × 10^2^	8.97 × 10^2^	8.84 × 10^2^
	Std	2.15 × 10^1^	2.38 × 10^1^	2.50 × 10^1^	1.91 × 10^1^	3.53 × 10^1^	1.21 × 10^1^	4.61 × 10^1^	2.26 × 10^1^	2.26 × 10^1^	4.71 × 10^1^	4.40 × 10^1^	3.41 × 10^1^	3.26 × 10^1^	1.12 × 10^1^	2.21 × 10^1^
	Rank	5	7	2	6	13	1	10	9	15	14	12	11	8	4	3
F9	Min	1.51 × 10^4^	1.80 × 10^4^	1.58 × 10^4^	1.78 × 10^4^	2.13 × 10^4^	1.62 × 10^4^	2.68 × 10^4^	2.47 × 10^4^	3.21 × 10^4^	2.51 × 10^4^	2.21 × 10^4^	2.10 × 10^4^	1.92 × 10^4^	1.96 × 10^4^	2.41 × 10^4^
	Mean	1.80 × 10^4^	2.87 × 10^4^	**1.77 × 10^4^**	2.02 × 10^4^	2.45 × 10^4^	2.79 × 10^4^	3.08 × 10^4^	2.79 × 10^4^	3.32 × 10^4^	3.07 × 10^4^	2.60 × 10^4^	2.31 × 10^4^	2.25 × 10^4^	2.38 × 10^4^	2.89 × 10^4^
	Std	1.53 × 10^3^	4.55 × 10^3^	1.02 × 10^3^	1.36 × 10^3^	1.62 × 10^3^	6.81 × 10^3^	1.88 × 10^3^	1.92 × 10^3^	4.86 × 10^2^	2.27 × 10^3^	2.02 × 10^3^	1.39 × 10^3^	1.98 × 10^3^	1.95 × 10^3^	1.78 × 10^3^
	Rank	2	11	1	3	7	9	14	10	15	13	8	5	4	6	12
F10	Min	2.56 × 10^3^	1.90 × 10^5^	9.63 × 10^4^	3.38 × 10^5^	9.79 × 10^8^	4.83 × 10^5^	7.83 × 10^5^	7.21 × 10^6^	5.08 × 10^7^	9.43 × 10^6^	5.26 × 10^5^	6.77 × 10^5^	3.62 × 10^5^	2.31 × 10^5^	4.72 × 10^5^
	Mean	**2.54 × 10^4^**	4.19 × 10^5^	6.64 × 10^5^	4.35 × 10^5^	4.51 × 10^9^	4.02 × 10^6^	8.11 × 10^8^	7.83 × 10^7^	1.32 × 10^8^	7.40 × 10^8^	1.24 × 10^6^	4.50 × 10^8^	7.13 × 10^5^	1.38 × 10^6^	7.26 × 10^5^
	Std	3.75 × 10^4^	1.89 × 10^5^	1.46 × 10^6^	6.89 × 10^4^	2.67 × 10^9^	5.01 × 10^6^	1.87 × 10^9^	6.32 × 10^7^	3.66 × 10^7^	8.36 × 10^8^	9.33 × 10^5^	1.40 × 10^9^	4.71 × 10^5^	1.82 × 10^6^	2.22 × 10^5^
	Rank	1	2	4	3	15	9	14	10	11	13	7	12	5	8	6
F11	Min	6.43 × 10^5^	1.66 × 10^9^	2.93 × 10^8^	1.46 × 10^8^	6.73 × 10^10^	1.08 × 10^9^	1.03 × 10^10^	2.58 × 10^10^	3.62 × 10^10^	6.28 × 10^10^	2.33 × 10^9^	2.68 × 10^9^	7.27 × 10^8^	1.07 × 10^9^	9.71 × 10^8^
	Mean	**2.15 × 10^6^**	4.89 × 10^9^	5.50 × 10^8^	3.46 × 10^8^	1.21 × 10^11^	6.42 × 10^9^	5.45 × 10^10^	4.24 × 10^10^	4.58 × 10^10^	8.97 × 10^10^	4.63 × 10^9^	1.76 × 10^10^	1.73 × 10^9^	2.00 × 10^9^	1.90 × 10^9^
	Std	1.06 × 10^6^	4.29 × 10^9^	2.01 × 10^8^	1.41 × 10^8^	3.35 × 10^10^	3.56 × 10^9^	4.34 × 10^10^	9.78 × 10^9^	3.99 × 10^9^	1.85 × 10^10^	1.60 × 10^9^	1.58 × 10^10^	6.31 × 10^8^	6.96 × 10^8^	6.23 × 10^8^
	Rank	1	8	3	2	15	9	13	11	12	14	7	10	4	6	5
F12	Min	2.28 × 10^3^	2.21 × 10^8^	9.52 × 10^7^	1.98 × 10^7^	1.06 × 10^11^	1.34 × 10^9^	1.05 × 10^10^	3.09 × 10^10^	6.82 × 10^10^	8.77 × 10^10^	1.44 × 10^9^	3.09 × 10^9^	2.19 × 10^8^	6.82 × 10^8^	4.67 × 10^8^
	Mean	**2.17 × 10^4^**	4.09 × 10^9^	2.92 × 10^8^	5.67 × 10^7^	2.11 × 10^11^	7.57 × 10^9^	4.28 × 10^10^	7.68 × 10^10^	8.05 × 10^10^	1.48 × 10^11^	2.85 × 10^9^	3.12 × 10^10^	6.69 × 10^8^	1.70 × 10^9^	1.15 × 10^9^
	Std	2.48 × 10^4^	4.23 × 10^9^	1.01 × 10^8^	3.93 × 10^7^	6.12 × 10^10^	4.22 × 10^9^	4.57 × 10^10^	2.53 × 10^10^	7.74 × 10^9^	3.55 × 10^10^	9.45 × 10^8^	4.77 × 10^10^	2.87 × 10^8^	9.45 × 10^8^	5.15 × 10^8^
	Rank	1	8	3	2	15	9	11	12	13	14	7	10	4	6	5
F13	Min	5.85 × 10^5^	4.95 × 10^5^	3.14 × 10^6^	8.99 × 10^5^	2.73 × 10^7^	1.17 × 10^6^	4.68 × 10^6^	1.43 × 10^7^	5.45 × 10^7^	6.13 × 10^6^	2.80 × 10^6^	2.57 × 10^6^	1.25 × 10^6^	4.90 × 10^6^	1.49 × 10^6^
	Mean	**3.09 × 10^6^**	7.65 × 10^6^	2.24 × 10^7^	6.77 × 10^6^	7.48 × 10^7^	4.75 × 10^6^	2.59 × 10^8^	4.87 × 10^7^	1.04 × 10^8^	3.28 × 10^7^	1.84 × 10^7^	1.42 × 10^7^	9.83 × 10^6^	1.63 × 10^7^	9.58 × 10^6^
	Std	2.63 × 10^6^	5.03 × 10^6^	9.62 × 10^6^	3.17 × 10^6^	3.82 × 10^7^	2.43 × 10^6^	1.95 × 10^8^	2.51 × 10^7^	2.36 × 10^7^	2.23 × 10^7^	9.59 × 10^6^	8.47 × 10^6^	5.46 × 10^6^	7.61 × 10^6^	4.19 × 10^6^
	Rank	1	4	10	3	13	2	15	12	14	11	9	7	6	8	5
F14	Min	7.14 × 10^3^	2.89 × 10^6^	2.01 × 10^7^	8.75 × 10^5^	2.52 × 10^10^	3.71 × 10^7^	6.33 × 10^8^	2.40 × 10^9^	1.61 × 10^10^	7.37 × 10^9^	1.80 × 10^8^	7.04 × 10^7^	3.44 × 10^7^	2.89 × 10^7^	2.30 × 10^7^
	Mean	**1.53 × 10^5^**	1.69 × 10^8^	4.39 × 10^7^	5.41 × 10^6^	3.92 × 10^10^	7.92 × 10^8^	1.04 × 10^10^	6.87 × 10^9^	2.06 × 10^10^	2.08 × 10^10^	3.62 × 10^8^	1.97 × 10^9^	7.38 × 10^7^	1.61 × 10^8^	9.70 × 10^7^
	Std	1.51 × 10^5^	3.20 × 10^8^	1.52 × 10^7^	5.49 × 10^6^	9.55 × 10^9^	9.27 × 10^8^	1.14 × 10^10^	2.09 × 10^9^	2.55 × 10^9^	7.78 × 10^9^	1.04 × 10^8^	3.33 × 10^9^	2.93 × 10^7^	8.56 × 10^7^	4.06 × 10^7^
	Rank	1	7	3	2	15	9	12	11	13	14	8	10	4	6	5
F15	Min	1.54 × 10^3^	7.54 × 10^3^	2.94 × 10^4^	5.46 × 10^4^	1.69 × 10^9^	9.96 × 10^4^	6.41 × 10^5^	1.72 × 10^7^	1.35 × 10^8^	3.64 × 10^8^	3.00 × 10^5^	1.35 × 10^5^	7.63 × 10^4^	9.25 × 10^4^	7.47 × 10^4^
	Mean	**6.80 × 10^3^**	5.68 × 10^6^	2.36 × 10^5^	1.89 × 10^5^	6.43 × 10^9^	1.12 × 10^7^	1.34 × 10^7^	2.07 × 10^8^	2.89 × 10^8^	2.18 × 10^9^	1.21 × 10^6^	1.79 × 10^8^	1.29 × 10^5^	8.99 × 10^5^	2.98 × 10^5^
	Std	5.14 × 10^3^	1.58 × 10^7^	2.16 × 10^5^	1.64 × 10^5^	3.95 × 10^9^	2.12 × 10^7^	1.73 × 10^7^	1.74 × 10^8^	7.90 × 10^7^	1.41 × 10^9^	1.18 × 10^6^	6.25 × 10^8^	5.89 × 10^4^	5.96 × 10^5^	2.41 × 10^5^
	Rank	1	8	4	3	15	9	10	12	13	14	7	11	2	6	5
F16	Min	2.66 × 10^3^	1.52 × 10^4^	6.91 × 10^3^	9.66 × 10^4^	4.39 × 10^11^	8.64 × 10^4^	3.42 × 10^11^	5.70 × 10^10^	4.76 × 10^12^	3.98 × 10^11^	1.93 × 10^8^	1.79 × 10^5^	1.07 × 10^5^	2.31 × 10^5^	1.26 × 10^5^
	Mean	**3.94 × 10^3^**	7.23 × 10^9^	7.29 × 10^6^	1.47 × 10^5^	8.93 × 10^14^	3.26 × 10^7^	4.14 × 10^15^	1.05 × 10^13^	5.74 × 10^13^	3.45 × 10^13^	6.48 × 10^10^	1.01 × 10^9^	3.61 × 10^6^	3.59 × 10^8^	3.61 × 10^5^
	Std	7.76 × 10^2^	3.38 × 10^10^	2.81 × 10^7^	3.24 × 10^4^	2.82 × 10^15^	1.63 × 10^8^	9.79 × 10^15^	1.72 × 10^13^	3.79 × 10^13^	3.47 × 10^13^	1.14 × 10^11^	3.66 × 10^9^	1.71 × 10^7^	8.33 × 10^8^	5.74 × 10^5^
	Rank	1	9	5	2	14	6	15	11	13	12	10	8	4	7	3
F17	Min	6.85 × 10^5^	1.91 × 10^6^	6.19 × 10^6^	2.55 × 10^6^	2.49 × 10^6^	9.50 × 10^5^	3.23 × 10^6^	3.84 × 10^6^	1.30 × 10^7^	5.01 × 10^6^	7.46 × 10^6^	1.42 × 10^6^	2.53 × 10^6^	2.24 × 10^6^	2.84 × 10^6^
	Mean	**4.82 × 10^6^**	1.41 × 10^7^	3.57 × 10^7^	9.07 × 10^6^	7.30 × 10^7^	4.98 × 10^6^	3.89 × 10^8^	6.16 × 10^7^	5.53 × 10^7^	4.24 × 10^7^	3.48 × 10^7^	3.43 × 10^7^	1.11 × 10^7^	1.89 × 10^7^	1.04 × 10^7^
	Std	3.63 × 10^6^	9.78 × 10^6^	2.52 × 10^7^	6.16 × 10^6^	8.13 × 10^7^	3.69 × 10^6^	4.48 × 10^8^	4.92 × 10^7^	1.98 × 10^7^	8.44 × 10^7^	2.16 × 10^7^	1.08 × 10^8^	8.92 × 10^6^	1.56 × 10^7^	6.33 × 10^6^
	Rank	1	6	10	3	14	2	15	13	12	11	9	8	5	7	4
F18	Min	2.10 × 10^3^	1.55 × 10^5^	2.11 × 10^7^	3.73 × 10^5^	1.25 × 10^13^	7.18 × 10^9^	3.01 × 10^10^	5.90 × 10^10^	5.55 × 10^10^	3.15 × 10^11^	3.08 × 10^10^	9.37 × 10^9^	8.97 × 10^9^	4.61 × 10^8^	3.76 × 10^8^
	Mean	**8.39 × 10^3^**	1.03 × 10^9^	1.25 × 10^8^	7.47 × 10^5^	1.02 × 10^15^	4.26 × 10^10^	1.06 × 10^14^	3.72 × 10^12^	9.66 × 10^10^	1.33 × 10^14^	9.38 × 10^10^	3.42 × 10^12^	3.17 × 10^10^	1.61 × 10^10^	3.03 × 10^9^
	Std	6.64 × 10^3^	1.28 × 10^9^	1.41 × 10^8^	5.87 × 10^5^	1.50 × 10^15^	7.58 × 10^10^	5.43 × 10^14^	9.98 × 10^12^	2.63 × 10^10^	2.70 × 10^14^	6.03 × 10^10^	1.60 × 10^13^	1.34 × 10^10^	2.56 × 10^10^	2.14 × 10^9^
	Rank	1	4	3	2	15	8	13	12	10	14	9	11	7	6	5
F19	Min	2.04 × 10^3^	3.35 × 10^3^	2.26 × 10^3^	4.02 × 10^3^	1.06 × 10^4^	3.77 × 10^3^	2.71 × 10^4^	2.14 × 10^4^	1.24 × 10^4^	2.61 × 10^4^	1.30 × 10^4^	6.67 × 10^3^	7.99 × 10^3^	7.02 × 10^3^	5.00 × 10^3^
	Mean	**2.48 × 10^3^**	1.14 × 10^4^	2.87 × 10^3^	6.29 × 10^3^	2.22 × 10^4^	5.47 × 10^3^	4.42 × 10^4^	2.82 × 10^4^	1.45 × 10^4^	3.66 × 10^4^	2.19 × 10^4^	1.25 × 10^4^	1.27 × 10^4^	9.45 × 10^3^	6.27 × 10^3^
	Std	3.81 × 10^2^	4.28 × 10^3^	3.43 × 10^2^	1.12 × 10^3^	6.91 × 10^3^	1.11 × 10^3^	5.65 × 10^3^	4.27 × 10^3^	1.00 × 10^3^	5.15 × 10^3^	6.27 × 10^3^	3.56 × 10^3^	2.64 × 10^3^	1.68 × 10^3^	9.96 × 10^2^
	Rank	1	7	2	5	12	3	15	13	10	14	11	8	9	6	4
F20	Min	2.10 × 10^3^	1.93 × 10^4^	4.64 × 10^3^	4.18 × 10^3^	1.78 × 10^5^	2.44 × 10^4^	1.36 × 10^5^	9.25 × 10^4^	1.84 × 10^5^	1.87 × 10^5^	1.04 × 10^4^	6.43 × 10^4^	2.14 × 10^4^	9.06 × 10^3^	1.03 × 10^4^
	Mean	**2.66 × 10^3^**	4.77 × 10^4^	5.33 × 10^3^	4.95 × 10^3^	2.97 × 10^5^	3.66 × 10^4^	1.85 × 10^5^	1.14 × 10^5^	2.28 × 10^5^	2.13 × 10^5^	3.32 × 10^4^	1.04 × 10^5^	3.15 × 10^4^	1.65 × 10^4^	1.50 × 10^4^
	Std	1.25 × 10^2^	1.47 × 10^4^	3.28 × 10^2^	3.62 × 10^2^	4.83 × 10^4^	7.91 × 10^3^	2.39 × 10^4^	1.11 × 10^4^	1.49 × 10^4^	1.44 × 10^4^	9.59 × 10^3^	3.46 × 10^4^	4.57 × 10^3^	3.30 × 10^3^	2.76 × 10^3^
	Rank	1	9	3	2	15	8	12	11	14	13	7	10	6	5	4
F21	Min	2.20 × 10^3^	2.89 × 10^3^	2.33 × 10^3^	2.34 × 10^3^	7.39 × 10^3^	2.54 × 10^3^	2.09 × 10^4^	1.69 × 10^4^	3.64 × 10^3^	2.05 × 10^4^	4.39 × 10^3^	3.24 × 10^3^	2.91 × 10^3^	2.67 × 10^3^	2.51 × 10^3^
	Mean	2.79 × 10^3^	4.69 × 10^3^	**2.36 × 10^3^**	4.34 × 10^3^	1.46 × 10^4^	2.71 × 10^3^	2.71 × 10^4^	2.09 × 10^4^	4.40 × 10^3^	2.81 × 10^4^	1.89 × 10^4^	1.44 × 10^4^	1.21 × 10^4^	6.67 × 10^3^	2.59 × 10^3^
	Std	2.57 × 10^3^	2.08 × 10^3^	7.91 × 10^0^	4.98 × 10^3^	3.18 × 10^3^	1.04 × 10^2^	3.36 × 10^3^	1.69 × 10^3^	1.03 × 10^3^	3.31 × 10^3^	6.88 × 10^3^	8.45 × 10^3^	6.98 × 10^3^	5.24 × 10^3^	4.82 × 10^2^
	Rank	4	7	1	5	11	3	14	13	6	15	12	10	9	8	2
F22	Min	2.30 × 10^3^	2.25 × 10^4^	4.58 × 10^3^	4.38 × 10^3^	1.25 × 10^5^	1.97 × 10^4^	1.07 × 10^5^	6.67 × 10^4^	6.55 × 10^4^	1.16 × 10^5^	1.99 × 10^4^	3.22 × 10^4^	2.82 × 10^4^	1.22 × 10^4^	1.08 × 10^4^
	Mean	**2.49 × 10^3^**	3.85 × 10^4^	7.59 × 10^3^	5.45 × 10^3^	1.73 × 10^5^	3.21 × 10^4^	1.18 × 10^5^	9.11 × 10^4^	8.35 × 10^4^	1.21 × 10^5^	3.13 × 10^4^	4.73 × 10^4^	4.63 × 10^4^	2.10 × 10^4^	1.68 × 10^4^
	Std	1.81 × 10^2^	1.66 × 10^4^	1.30 × 10^3^	5.73 × 10^2^	2.31 × 10^4^	6.98 × 10^3^	4.41 × 10^3^	7.49 × 10^3^	7.49 × 10^3^	3.38 × 10^3^	1.61 × 10^4^	9.81 × 10^3^	1.43 × 10^4^	1.33 × 10^4^	5.23 × 10^3^
	Rank	1	8	3	2	15	7	13	12	11	14	6	10	9	5	4
F23	Min	2.50 × 10^3^	2.98 × 10^4^	5.76 × 10^3^	5.28 × 10^3^	1.32 × 10^5^	3.01 × 10^4^	1.04 × 10^5^	9.39 × 10^4^	9.15 × 10^4^	1.44 × 10^5^	3.38 × 10^4^	3.77 × 10^4^	3.39 × 10^4^	2.10 × 10^4^	1.83 × 10^4^
	Mean	**2.59 × 10^3^**	5.87 × 10^4^	1.14 × 10^4^	7.58 × 10^3^	2.35 × 10^5^	4.56 × 10^4^	1.49 × 10^5^	1.15 × 10^5^	1.06 × 10^5^	1.60 × 10^5^	5.66 × 10^4^	6.92 × 10^4^	5.94 × 10^4^	3.40 × 10^4^	2.46 × 10^4^
	Std	2.13 × 10^2^	1.67 × 10^4^	1.78 × 10^3^	1.08 × 10^3^	3.57 × 10^4^	7.39 × 10^3^	1.49 × 10^4^	7.57 × 10^3^	7.61 × 10^3^	7.79 × 10^3^	1.92 × 10^4^	2.75 × 10^4^	1.60 × 10^4^	1.06 × 10^4^	3.80 × 10^3^
	Rank	1	8	3	2	15	6	13	12	11	14	7	10	9	5	4
F24	Min	3.28 × 10^3^	5.15 × 10^3^	3.65 × 10^3^	3.47 × 10^3^	2.37 × 10^4^	4.41 × 10^3^	8.79 × 10^3^	9.45 × 10^3^	2.08 × 10^4^	1.23 × 10^4^	5.17 × 10^3^	6.19 × 10^3^	4.69 × 10^3^	4.61 × 10^3^	4.33 × 10^3^
	Mean	**3.31 × 10^3^**	7.32 × 10^3^	3.93 × 10^3^	3.65 × 10^3^	3.65 × 10^4^	5.60 × 10^3^	1.67 × 10^4^	1.22 × 10^4^	2.78 × 10^4^	1.90 × 10^4^	6.22 × 10^3^	9.22 × 10^3^	5.73 × 10^3^	5.13 × 10^3^	4.83 × 10^3^
	Std	2.29 × 10^1^	1.68 × 10^3^	1.77 × 10^2^	9.45 × 10^1^	6.95 × 10^3^	5.10 × 10^2^	7.92 × 10^3^	1.50 × 10^3^	2.79 × 10^3^	2.78 × 10^3^	5.61 × 10^2^	2.03 × 10^3^	7.05 × 10^2^	3.17 × 10^2^	3.10 × 10^2^
	Rank	1	9	3	2	15	6	12	11	14	13	8	10	7	5	4
F25	Min	5.83 × 10^3^	1.93 × 10^4^	6.10 × 10^3^	6.17 × 10^3^	4.77 × 10^4^	7.86 × 10^3^	1.02 × 10^5^	3.94 × 10^4^	8.39 × 10^3^	3.24 × 10^4^	1.04 × 10^4^	9.76 × 10^3^	1.50 × 10^4^	6.53 × 10^3^	6.83 × 10^3^
	Mean	**5.97 × 10^3^**	3.45 × 10^4^	6.30 × 10^3^	6.52 × 10^3^	1.02 × 10^5^	9.70 × 10^3^	2.52 × 10^5^	1.08 × 10^5^	9.13 × 10^3^	8.44 × 10^4^	2.13 × 10^4^	3.07 × 10^4^	2.74 × 10^4^	7.15 × 10^3^	7.47 × 10^3^
	Std	5.21 × 10^1^	1.21 × 10^4^	9.86 × 10^1^	1.51 × 10^2^	3.16 × 10^4^	1.05 × 10^3^	6.74 × 10^4^	4.37 × 10^4^	4.05 × 10^2^	5.01 × 10^4^	7.03 × 10^3^	1.71 × 10^4^	9.09 × 10^3^	4.06 × 10^2^	3.51 × 10^2^
	Rank	1	11	2	3	13	7	15	14	6	12	8	10	9	4	5
F26	Min	3.31 × 10^3^	4.36 × 10^3^	3.38 × 10^3^	3.38 × 10^3^	5.54 × 10^3^	3.57 × 10^3^	7.67 × 10^3^	5.70 × 10^3^	5.01 × 10^3^	6.52 × 10^3^	4.36 × 10^3^	3.59 × 10^3^	3.73 × 10^3^	3.77 × 10^3^	3.56 × 10^3^
	Mean	**3.44 × 10^3^**	5.30 × 10^3^	3.55 × 10^3^	3.59 × 10^3^	6.94 × 10^3^	3.84 × 10^3^	1.01 × 10^4^	8.74 × 10^3^	5.30 × 10^3^	9.38 × 10^3^	5.43 × 10^3^	4.14 × 10^3^	4.31 × 10^3^	4.06 × 10^3^	3.88 × 10^3^
	Std	9.82 × 10^1^	5.01 × 10^2^	1.05 × 10^2^	1.04 × 10^2^	1.06 × 10^3^	1.41 × 10^2^	1.11 × 10^3^	1.52 × 10^3^	1.78 × 10^2^	1.23 × 10^3^	5.47 × 10^2^	3.57 × 10^2^	3.74 × 10^2^	3.17 × 10^2^	1.41 × 10^2^
	Rank	1	10	2	3	12	4	15	13	9	14	11	7	8	6	5
F27	Min	2.70 × 10^3^	4.19 × 10^3^	3.28 × 10^3^	3.21 × 10^3^	8.43 × 10^3^	3.68 × 10^3^	7.18 × 10^3^	7.62 × 10^3^	8.15 × 10^3^	9.09 × 10^3^	3.89 × 10^3^	4.37 × 10^3^	3.57 × 10^3^	3.50 × 10^3^	3.43 × 10^3^
	Mean	**2.72 × 10^3^**	6.15 × 10^3^	3.33 × 10^3^	3.28 × 10^3^	1.64 × 10^4^	4.09 × 10^3^	1.86 × 10^4^	9.50 × 10^3^	9.12 × 10^3^	1.32 × 10^4^	4.69 × 10^3^	6.49 × 10^3^	4.04 × 10^3^	3.66 × 10^3^	3.73 × 10^3^
	Std	9.62 × 10^0^	1.40 × 10^3^	2.43 × 10^1^	3.10 × 10^1^	4.31 × 10^3^	2.15 × 10^2^	6.90 × 10^3^	9.31 × 10^2^	5.97 × 10^2^	2.11 × 10^3^	4.12 × 10^2^	1.44 × 10^3^	2.97 × 10^2^	9.54 × 10^1^	1.59 × 10^2^
	Rank	1	9	3	2	14	7	15	12	11	13	8	10	6	4	5
F28	Min	1.06 × 10^4^	1.63 × 10^8^	2.45 × 10^6^	3.31 × 10^6^	2.65 × 10^13^	3.73 × 10^9^	3.63 × 10^10^	2.58 × 10^10^	7.58 × 10^11^	8.01 × 10^11^	1.53 × 10^10^	6.95 × 10^8^	3.45 × 10^9^	1.91 × 10^8^	1.03 × 10^9^
	Mean	**1.98 × 10^5^**	4.47 × 10^9^	1.81 × 10^10^	7.54 × 10^7^	2.11 × 10^15^	1.16 × 10^10^	7.82 × 10^13^	3.55 × 10^12^	8.75 × 10^12^	4.30 × 10^14^	7.78 × 10^10^	2.98 × 10^13^	1.61 × 10^10^	4.48 × 10^10^	3.21 × 10^9^
	Std	2.06 × 10^5^	5.13 × 10^9^	5.95 × 10^10^	1.25 × 10^8^	2.73 × 10^15^	6.13 × 10^9^	2.70 × 10^14^	4.89 × 10^12^	7.20 × 10^12^	8.98 × 10^14^	6.56 × 10^10^	1.35 × 10^14^	7.38 × 10^9^	1.73 × 10^11^	2.41 × 10^9^
	Rank	1	4	7	2	15	5	13	10	11	14	9	12	6	8	3
F29	Min	4.44 × 10^5^	2.10 × 10^8^	9.36 × 10^7^	5.01 × 10^6^	4.73 × 10^13^	7.80 × 10^9^	2.40 × 10^10^	6.44 × 10^10^	1.19 × 10^12^	2.24 × 10^12^	3.93 × 10^10^	2.05 × 10^10^	1.44 × 10^10^	1.11 × 10^9^	2.16 × 10^9^
	Mean	**9.96 × 10^5^**	5.61 × 10^9^	3.55 × 10^8^	5.60 × 10^7^	1.47 × 10^15^	2.39 × 10^10^	4.05 × 10^13^	1.73 × 10^12^	4.20 × 10^12^	1.52 × 10^14^	1.37 × 10^11^	3.73 × 10^14^	3.51 × 10^10^	1.17 × 10^10^	7.37 × 10^9^
	Std	4.32 × 10^5^	6.72 × 10^9^	2.37 × 10^8^	4.76 × 10^7^	1.83 × 10^15^	1.25 × 10^10^	1.70 × 10^14^	4.64 × 10^12^	2.10 × 10^12^	1.85 × 10^14^	9.89 × 10^10^	1.99 × 10^15^	1.26 × 10^10^	7.04 × 10^9^	4.84 × 10^9^
	Rank	1	4	3	2	15	7	12	10	11	13	9	14	8	6	5
**Total**		26	0	2	0	0	1	0	0	0	0	0	0	0	0	0

**Table 3:** Wilcoxon rank-sum test (Dim = 30). Bold *p*-values indicate statistically significant differences (*p* < 0.05).

Dim = 30														
Func.	PSO	BBO	SMA	DE	GWO	SSA	HHO	ABC	WOA	E-WOA	IWOA	IWOSSA	RAV-WOA	WOAAD
F1	**3.02 × 10^−11^**	**3.02 × 10^−11^**	**3.02 × 10^−11^**	**3.02 × 10^−11^**	**3.02 × 10^−11^**	**3.02 × 10^−11^**	**3.02 × 10^−11^**	**3.02 × 10^−11^**	**3.02 × 10^−11^**	**3.02 × 10^−11^**	**3.02 × 10^−11^**	**1.41 × 10^−9^**	**3.02 × 10^−11^**	**3.02 × 10^−11^**
	+	+	+	+	+	+	+	+	+	+	+	+	+	+
F2	**3.02 × 10^−11^**	**3.02 × 10^−11^**	**3.02 × 10^−11^**	**3.02 × 10^−11^**	**3.02 × 10^−11^**	**3.02 × 10^−11^**	**3.02 × 10^−11^**	**3.02 × 10^−11^**	**3.02 × 10^−11^**	**3.02 × 10^−11^**	**3.02 × 10^−11^**	**3.02 × 10^−11^**	**3.02 × 10^−11^**	**3.02 × 10^−11^**
	+	+	+	+	+	+	+	+	+	+	+	+	+	+
F3	**4.31 × 10^−8^**	**2.23 × 10^−9^**	**3.67 × 10^−3^**	**3.02 × 10^−11^**	**6.72 × 10^−10^**	**3.02 × 10^−11^**	**3.02 × 10^−11^**	**3.02 × 10^−11^**	**3.02 × 10^−11^**	**6.69 × 10^−11^**	**1.20 × 10^−10^**	**1.19 × 10^−6^**	**1.96 × 10^−10^**	**1.41 × 10^−9^**
	+	+	+	+	+	+	+	+	+	+	+	+	+	+
F4	**3.02 × 10^−11^**	**3.02 × 10^−11^**	**6.70 × 10^−11^**	**3.02 × 10^−11^**	**3.02 × 10^−11^**	**3.02 × 10^−11^**	**3.02 × 10^−11^**	**3.02 × 10^−11^**	**3.02 × 10^−11^**	**3.02 × 10^−11^**	**3.02 × 10^−11^**	**4.50 × 10^−11^**	**3.02 × 10^−11^**	**3.02 × 10^−11^**
	+	+	+	+	+	+	+	+	+	+	+	+	+	+
F5	**3.56 × 10^−4^**	**5.26 × 10^−4^**	**3.02 × 10^−11^**	**1.21 × 10^−10^**	5.01 × 10^0^	**2.44 × 10^−9^**	**3.47 × 10^−10^**	**3.02 × 10^−11^**	**6.70 × 10^−11^**	**8.89 × 10^−10^**	**7.60 × 10^−7^**	**9.21 × 10^−5^**	**1.02 × 10^−5^**	**1.89 × 10^−4^**
	+	+	+	+	=	+	+	+	+	+	+	+	+	+
F6	**6.70 × 10^−11^**	**1.41 × 10^−9^**	**3.08 × 10^−8^**	**1.31 × 10^−8^**	**3.02 × 10^−11^**	**4.57 × 10^−9^**	**3.02 × 10^−11^**	**3.02 × 10^−11^**	**6.70 × 10^−11^**	**4.98 × 10^−11^**	**1.78 × 10^−10^**	**1.87 × 10^−7^**	**2.87 × 10^−10^**	**8.15 × 10^−11^**
	+	+	+	+	+	+	+	+	+	+	+	+	+	+
F7	**6.91 × 10^−4^**	**1.68 × 10^−3^**	**2.75 × 10^−3^**	**4.74 × 10^−6^**	**1.68 × 10^−4^**	**6.74 × 10^−6^**	**1.07 × 10^−7^**	**3.34 × 10^−11^**	**3.35 × 10^−8^**	**1.43 × 10^−8^**	**9.51 × 10^−6^**	**2.05 × 10^−3^**	**2.42 × 10^−2^**	**8.15 × 10^−5^**
	+	+	+	+	+	+	+	+	+	+	+	+	+	+
F8	**1.09 × 10^−5^**	**2.87 × 10^−10^**	**2.96 × 10^−5^**	5.94 × 10^0^	**1.78 × 10^−10^**	**8.31 × 10^−3^**	1.76 × 10^−1^	**4.64 × 10^−3^**	**3.85 × 10^−3^**	1.45 × 10^−1^	2.01 × 10^−1^	**5.08 × 10^−3^**	**5.19 × 10^−7^**	**5.07 × 10^−10^**
	−	−	−	=	−	−	=	+	+	=	=	−	−	−
F9	**2.05 × 10^−3^**	**6.53 × 10^−8^**	**1.44 × 10^−3^**	3.11 × 10^−1^	5.30 × 10^−1^	**1.25 × 10^−7^**	**4.64 × 10^−5^**	**3.02 × 10^−11^**	**1.29 × 10^−6^**	**5.87 × 10^−4^**	1.02 × 10^−1^	1.05 × 10^−1^	9.33 × 10^−2^	5.49 × 10^−1^
	−	−	−	=	=	+	+	+	+	+	=	=	=	=
F10	6.41 × 10^0^	**8.31 × 10^−3^**	**2.68 × 10^−4^**	**3.02 × 10^−11^**	**3.16 × 10^−10^**	**1.78 × 10^−10^**	**6.70 × 10^−11^**	**3.02 × 10^−11^**	**9.76 × 10^−10^**	**3.02 × 10^−11^**	**1.86 × 10^−9^**	**4.08 × 10^−11^**	6.35 × 10^−2^	**9.26 × 10^−9^**
	=	+	+	+	+	+	+	+	+	+	+	+	=	+
F11	9.82 × 10^0^	**3.02 × 10^−11^**	**9.92 × 10^−11^**	**3.02 × 10^−11^**	**3.02 × 10^−11^**	**3.02 × 10^−11^**	**3.02 × 10^−11^**	**3.02 × 10^−11^**	**3.02 × 10^−11^**	**3.02 × 10^−11^**	**3.02 × 10^−11^**	**3.02 × 10^−11^**	**3.02 × 10^−11^**	**3.02 × 10^−11^**
	=	+	+	+	+	+	+	+	+	+	+	+	+	+
F12	**3.09 × 10^−6^**	**3.02 × 10^−11^**	**3.02 × 10^−11^**	**3.02 × 10^−11^**	**3.02 × 10^−11^**	**3.02 × 10^−11^**	**3.02 × 10^−11^**	**3.02 × 10^−11^**	**3.02 × 10^−11^**	**3.02 × 10^−11^**	**3.02 × 10^−11^**	**3.02 × 10^−11^**	**3.02 × 10^−11^**	**3.02 × 10^−11^**
	+	+	+	+	+	+	+	+	+	+	+	+	+	+
F13	**2.42 × 10^−2^**	**1.53 × 10^−5^**	7.96 × 10^−1^	**2.23 × 10^−9^**	4.92 × 10^−1^	**9.76 × 10^−10^**	**1.55 × 10^−9^**	**2.19 × 10^−8^**	**1.24 × 10^−3^**	**7.04 × 10^−7^**	**7.96 × 10^−3^**	**1.30 × 10^−3^**	1.96 × 10^−1^	3.87 × 10^−1^
	−	+	=	+	=	+	+	+	+	+	+	+	=	=
F14	1.22 × 10^−1^	**3.02 × 10^−11^**	**3.34 × 10^−11^**	**3.02 × 10^−11^**	**3.02 × 10^−11^**	**3.02 × 10^−11^**	**3.02 × 10^−11^**	**3.02 × 10^−11^**	**3.02 × 10^−11^**	**3.02 × 10^−11^**	**3.69 × 10^−11^**	**3.02 × 10^−11^**	**5.49 × 10^−11^**	**3.02 × 10^−11^**
	=	+	+	+	+	+	+	+	+	+	+	+	+	+
F15	2.12 × 10^−1^	**3.02 × 10^−11^**	**1.29 × 10^−9^**	**3.02 × 10^−11^**	**3.02 × 10^−11^**	**3.34 × 10^−11^**	**3.02 × 10^−11^**	**4.50 × 10^−11^**	**3.02 × 10^−11^**	**3.02 × 10^−11^**	**3.69 × 10^−11^**	**3.02 × 10^−11^**	**3.34 × 10^−11^**	**3.02 × 10^−11^**
	=	+	+	+	+	+	+	+	+	+	+	+	+	+
F16	**2.87 × 10^−10^**	**8.99 × 10^−11^**	**3.16 × 10^−10^**	**3.02 × 10^−11^**	**3.02 × 10^−11^**	**3.02 × 10^−11^**	**3.02 × 10^−11^**	**3.02 × 10^−11^**	**3.02 × 10^−11^**	**3.02 × 10^−11^**	**3.02 × 10^−11^**	**3.02 × 10^−11^**	**3.69 × 10^−11^**	**3.02 × 10^−11^**
	+	+	+	+	+	+	+	+	+	+	+	+	+	+
F17	**2.75 × 10^−3^**	**7.38 × 10^−10^**	**3.32 × 10^−6^**	**6.52 × 10^−9^**	**1.75 × 10^−5^**	**1.37 × 10^−3^**	**3.65 × 10^−8^**	**8.15 × 10^−11^**	**9.03 × 10^−4^**	**4.18 × 10^−9^**	**5.61 × 10^−5^**	**5.07 × 10^−10^**	**3.27 × 10^−2^**	**1.16 × 10^−7^**
	+	+	+	+	+	+	+	+	+	+	+	+	+	+
F18	6.00 × 10^0^	**3.02 × 10^−11^**	**8.84 × 10^−7^**	**3.02 × 10^−11^**	**9.76 × 10^−10^**	**4.50 × 10^−11^**	**3.02 × 10^−11^**	**3.02 × 10^−11^**	**3.02 × 10^−11^**	**3.02 × 10^−11^**	**5.49 × 10^−11^**	**8.89 × 10^−10^**	**1.46 × 10^−10^**	**5.49 × 10^−11^**
	=	+	+	+	+	+	+	+	+	+	+	+	+	+
F19	**6.53 × 10^−8^**	4.55 × 10^0^	4.46 × 10^0^	**3.02 × 10^−11^**	**1.00 × 10^−3^**	**3.02 × 10^−11^**	**3.02 × 10^−11^**	**3.02 × 10^−11^**	**3.02 × 10^−11^**	**3.02 × 10^−11^**	**5.07 × 10^−10^**	**3.02 × 10^−11^**	**4.57 × 10^−9^**	**2.38 × 10^−3^**
	+	=	=	+	+	+	+	+	+	+	+	+	+	+
F20	**4.11 × 10^−7^**	**2.37 × 10^−10^**	2.97 × 10^0^	**3.02 × 10^−11^**	**3.02 × 10^−11^**	**3.02 × 10^−11^**	**3.02 × 10^−11^**	**3.02 × 10^−11^**	**3.02 × 10^−11^**	**2.61 × 10^−10^**	**3.02 × 10^−11^**	**1.56 × 10^−2^**	**2.15 × 10^−6^**	**7.38 × 10^−10^**
	+	+	=	+	+	+	+	+	+	+	+	+	+	+
F21	**1.70 × 10^−8^**	1.71 × 10^−1^	5.01 × 10^−2^	**3.02 × 10^−11^**	**2.89 × 10^−3^**	**3.02 × 10^−11^**	**3.02 × 10^−11^**	**9.76 × 10^−10^**	**3.02 × 10^−11^**	**3.69 × 10^−11^**	**3.20 × 10^−9^**	**9.53 × 10^−7^**	**4.03 × 10^−3^**	**5.32 × 10^−3^**
	+	=	=	+	+	+	+	+	+	+	+	+	+	+
F22	**1.09 × 10^−10^**	**3.02 × 10^−11^**	**1.24 × 10^−3^**	**3.02 × 10^−11^**	**3.02 × 10^−11^**	**3.02 × 10^−11^**	**3.02 × 10^−11^**	**3.02 × 10^−11^**	**3.02 × 10^−11^**	**3.02 × 10^−11^**	**3.02 × 10^−11^**	**3.18 × 10^−3^**	**3.02 × 10^−11^**	**3.02 × 10^−11^**
	+	+	+	+	+	+	+	+	+	+	+	+	+	+
F23	**3.96 × 10^−8^**	**1.31 × 10^−8^**	**6.74 × 10^−6^**	**3.02 × 10^−11^**	**8.10 × 10^−10^**	**3.02 × 10^−11^**	**3.02 × 10^−11^**	**3.02 × 10^−11^**	**3.02 × 10^−11^**	**7.12 × 10^−9^**	**5.49 × 10^−11^**	**6.28 × 10^−6^**	**1.56 × 10^−8^**	**2.60 × 10^−8^**
	+	+	+	+	+	+	+	+	+	+	+	+	+	+
F24	**2.15 × 10^−10^**	**1.33 × 10^−10^**	4.55 × 10^0^	**3.02 × 10^−11^**	**3.34 × 10^−11^**	**3.02 × 10^−11^**	**3.02 × 10^−11^**	**3.02 × 10^−11^**	**3.02 × 10^−11^**	**3.02 × 10^−11^**	**4.08 × 10^−11^**	**4.50 × 10^−11^**	**2.61 × 10^−10^**	**4.20 × 10^−10^**
	+	+	=	+	+	+	+	+	+	+	+	+	+	+
F25	**5.57 × 10^−10^**	**3.02 × 10^−11^**	**3.69 × 10^−11^**	**3.02 × 10^−11^**	**3.02 × 10^−11^**	**3.02 × 10^−11^**	**3.02 × 10^−11^**	**3.02 × 10^−11^**	**3.02 × 10^−11^**	**3.02 × 10^−11^**	**3.02 × 10^−11^**	**3.02 × 10^−11^**	**3.02 × 10^−11^**	**3.02 × 10^−11^**
	+	+	+	+	+	+	+	+	+	+	+	+	+	+
F26	**7.12 × 10^−9^**	2.51 × 10^−2^	3.18 × 10^−3^	**3.02 × 10^−11^**	1.56 × 10^−2^	**3.02 × 10^−11^**	**3.02 × 10^−11^**	4.06 × 10^−2^	**3.02 × 10^−11^**	**8.48 × 10^−9^**	**2.53 × 10^−4^**	**1.43 × 10^−5^**	**1.91 × 10^−2^**	**7.96 × 10^−3^**
	+	−	−	+	−	+	+	+	+	+	+	+	+	−
F27	**3.02 × 10^−11^**	**3.02 × 10^−11^**	**3.02 × 10^−11^**	**3.02 × 10^−11^**	**3.02 × 10^−11^**	**3.02 × 10^−11^**	**3.02 × 10^−11^**	**3.02 × 10^−11^**	**3.02 × 10^−11^**	**3.02 × 10^−11^**	**3.02 × 10^−11^**	**3.02 × 10^−11^**	**3.02 × 10^−11^**	**3.02 × 10^−11^**
	+	+	+	+	+	+	+	+	+	+	+	+	+	+
F28	**1.20 × 10^−8^**	**5.49 × 10^−11^**	**1.86 × 10^−6^**	**3.02 × 10^−11^**	**1.17 × 10^−9^**	**3.02 × 10^−11^**	**3.02 × 10^−11^**	**3.02 × 10^−11^**	**3.02 × 10^−11^**	**9.92 × 10^−11^**	**8.15 × 10^−11^**	**2.61 × 10^−10^**	**2.44 × 10^−9^**	**3.82 × 10^−9^**
	+	+	+	+	+	+	+	+	+	+	+	+	+	+
F29	**6.05 × 10^−7^**	**3.02 × 10^−11^**	**2.37 × 10^−10^**	**3.02 × 10^−11^**	**3.02 × 10^−11^**	**3.02 × 10^−11^**	**3.02 × 10^−11^**	**3.02 × 10^−11^**	**3.02 × 10^−11^**	**3.02 × 10^−11^**	**2.87 × 10^−10^**	**3.02 × 10^−11^**	**3.69 × 10^−11^**	**3.02 × 10^−11^**
	+	+	+	+	+	+	+	+	+	+	+	+	+	+

**Table 4:** Wilcoxon rank-sum test (Dim = 100). Bold *p*-values indicate statistically significant differences (*p* < 0.05).

Dim = 100														
Func.	PSO	BBO	SMA	DE	GWO	SSA	HHO	ABC	WOA	E-WOA	IWOA	IWOSSA	RAV-WOA	WOAAD
F1	**3.02 × 10^−11^**	**3.02 × 10^−11^**	**3.02 × 10^−11^**	**3.02 × 10^−11^**	**3.02 × 10^−11^**	**3.02 × 10^−11^**	**3.02 × 10^−11^**	**3.02 × 10^−11^**	**3.02 × 10^−11^**	**3.02 × 10^−11^**	**3.02 × 10^−11^**	**3.02 × 10^−11^**	**3.02 × 10^−11^**	**3.02 × 10^−11^**
	+	+	+	+	+	+	+	+	+	+	+	+	+	+
F2	**3.02 × 10^−11^**	**3.02 × 10^−11^**	**1.87 × 10^−5^**	**3.02 × 10^−11^**	**3.02 × 10^−11^**	**3.02 × 10^−11^**	**3.02 × 10^−11^**	**3.02 × 10^−11^**	**3.02 × 10^−11^**	**3.02 × 10^−11^**	**3.02 × 10^−11^**	**3.02 × 10^−11^**	**3.02 × 10^−11^**	**3.02 × 10^−11^**
	+	+	+	+	+	+	+	+	+	+	+	+	+	+
F3	**3.02 × 10^−11^**	**3.02 × 10^−11^**	**3.02 × 10^−11^**	**3.02 × 10^−11^**	**3.02 × 10^−11^**	**3.02 × 10^−11^**	**3.02 × 10^−11^**	**3.02 × 10^−11^**	**3.02 × 10^−11^**	**3.02 × 10^−11^**	**3.02 × 10^−11^**	**3.02 × 10^−11^**	**3.02 × 10^−11^**	**3.02 × 10^−11^**
	+	+	+	+	+	+	+	+	+	+	+	+	+	+
F4	**3.02 × 10^−11^**	**3.02 × 10^−11^**	**3.02 × 10^−11^**	**3.02 × 10^−11^**	**3.02 × 10^−11^**	**3.02 × 10^−11^**	**3.02 × 10^−11^**	**3.02 × 10^−11^**	**3.02 × 10^−11^**	**3.02 × 10^−11^**	**3.02 × 10^−11^**	**3.02 × 10^−11^**	**3.02 × 10^−11^**	**3.02 × 10^−11^**
	+	+	+	+	+	+	+	+	+	+	+	+	+	+
F5	**2.37 × 10^−10^**	**2.03 × 10^−9^**	**3.02 × 10^−11^**	**3.02 × 10^−11^**	**2.20 × 10^−7^**	**3.02 × 10^−11^**	**3.02 × 10^−11^**	**3.02 × 10^−11^**	**3.02 × 10^−11^**	**3.02 × 10^−11^**	**3.02 × 10^−11^**	**6.70 × 10^−11^**	**3.82 × 10^−10^**	**3.69 × 10^−11^**
	+	+	+	+	+	+	+	+	+	+	+	+	+	+
F6	**3.02 × 10^−11^**	**3.02 × 10^−11^**	**3.02 × 10^−11^**	**3.02 × 10^−11^**	**3.02 × 10^−11^**	**3.02 × 10^−11^**	**3.02 × 10^−11^**	**3.02 × 10^−11^**	**3.02 × 10^−11^**	**3.02 × 10^−11^**	**3.02 × 10^−11^**	**3.02 × 10^−11^**	**3.02 × 10^−11^**	**3.02 × 10^−11^**
	+	+	+	+	+	+	+	+	+	+	+	+	+	+
F7	**3.34 × 10^−11^**	**1.61 × 10^−10^**	**3.69 × 10^−11^**	**3.02 × 10^−11^**	**1.96 × 10^−10^**	**3.02 × 10^−11^**	**3.02 × 10^−11^**	**3.02 × 10^−11^**	**3.02 × 10^−11^**	**3.02 × 10^−11^**	**3.02 × 10^−11^**	**1.09 × 10^−10^**	**1.46 × 10^−10^**	**3.02 × 10^−11^**
	+	+	+	+	+	+	+	+	+	+	+	+	+	+
F8	3.87 × 10^0^	**2.49 × 10^−6^**	3.63 × 10^0^	**3.02 × 10^−11^**	**2.15 × 10^−10^**	**1.86 × 10^−9^**	**1.55 × 10^−9^**	**3.02 × 10^−11^**	**3.02 × 10^−11^**	**4.98 × 10^−11^**	**1.61 × 10^−10^**	**9.21 × 10^−5^**	6.41 × 10^0^	**6.97 × 10^−3^**
	=	−	=	+	−	+	+	+	+	+	+	+	=	−
F9	**1.78 × 10^−10^**	3.79 × 10^0^	**3.83 × 10^−6^**	**3.02 × 10^−11^**	**4.44 × 10^−7^**	**3.02 × 10^−11^**	**3.02 × 10^−11^**	**3.02 × 10^−11^**	**3.02 × 10^−11^**	**3.02 × 10^−11^**	**3.02 × 10^−11^**	**2.87 × 10^−10^**	**6.07 × 10^−11^**	**3.02 × 10^−11^**
	+	=	+	+	+	+	+	+	+	+	+	+	+	+
F10	**3.02 × 10^−11^**	**8.99 × 10^−11^**	**3.02 × 10^−11^**	**3.02 × 10^−11^**	**3.02 × 10^−11^**	**3.02 × 10^−11^**	**3.02 × 10^−11^**	**3.02 × 10^−11^**	**3.02 × 10^−11^**	**3.02 × 10^−11^**	**3.02 × 10^−11^**	**3.02 × 10^−11^**	**3.02 × 10^−11^**	**3.02 × 10^−11^**
	+	+	+	+	+	+	+	+	+	+	+	+	+	+
F11	**3.02 × 10^−11^**	**3.02 × 10^−11^**	**3.02 × 10^−11^**	**3.02 × 10^−11^**	**3.02 × 10^−11^**	**3.02 × 10^−11^**	**3.02 × 10^−11^**	**3.02 × 10^−11^**	**3.02 × 10^−11^**	**3.02 × 10^−11^**	**3.02 × 10^−11^**	**3.02 × 10^−11^**	**3.02 × 10^−11^**	**3.02 × 10^−11^**
	+	+	+	+	+	+	+	+	+	+	+	+	+	+
F12	**3.02 × 10^−11^**	**3.02 × 10^−11^**	**3.02 × 10^−11^**	**3.02 × 10^−11^**	**3.02 × 10^−11^**	**3.02 × 10^−11^**	**3.02 × 10^−11^**	**3.02 × 10^−11^**	**3.02 × 10^−11^**	**3.02 × 10^−11^**	**3.02 × 10^−11^**	**3.02 × 10^−11^**	**3.02 × 10^−11^**	**3.02 × 10^−11^**
	+	+	+	+	+	+	+	+	+	+	+	+	+	+
F13	**2.60 × 10^−5^**	**1.46 × 10^−10^**	**1.02 × 10^−5^**	**3.02 × 10^−11^**	**2.05 × 10^−3^**	**7.39 × 10^−11^**	**3.02 × 10^−11^**	**3.02 × 10^−11^**	**6.07 × 10^−11^**	**5.57 × 10^−10^**	**2.44 × 10^−9^**	**1.47 × 10^−7^**	**3.82 × 10^−10^**	**3.96 × 10^−8^**
	+	+	+	+	+	+	+	+	+	+	+	+	+	+
F14	**3.02 × 10^−11^**	**3.02 × 10^−11^**	**3.02 × 10^−11^**	**3.02 × 10^−11^**	**3.02 × 10^−11^**	**3.02 × 10^−11^**	**3.02 × 10^−11^**	**3.02 × 10^−11^**	**3.02 × 10^−11^**	**3.02 × 10^−11^**	**3.02 × 10^−11^**	**3.02 × 10^−11^**	**3.02 × 10^−11^**	**3.02 × 10^−11^**
	+	+	+	+	+	+	+	+	+	+	+	+	+	+
F15	**3.16 × 10^−10^**	**3.02 × 10^−11^**	**3.02 × 10^−11^**	**3.02 × 10^−11^**	**3.02 × 10^−11^**	**3.02 × 10^−11^**	**3.02 × 10^−11^**	**3.02 × 10^−11^**	**3.02 × 10^−11^**	**3.02 × 10^−11^**	**3.02 × 10^−11^**	**3.02 × 10^−11^**	**3.02 × 10^−11^**	**3.02 × 10^−11^**
	+	+	+	+	+	+	+	+	+	+	+	+	+	+
F16	**3.02 × 10^−11^**	**3.02 × 10^−11^**	**3.02 × 10^−11^**	**3.02 × 10^−11^**	**3.02 × 10^−11^**	**3.02 × 10^−11^**	**3.02 × 10^−11^**	**3.02 × 10^−11^**	**3.02 × 10^−11^**	**3.02 × 10^−11^**	**3.02 × 10^−11^**	**3.02 × 10^−11^**	**3.02 × 10^−11^**	**3.02 × 10^−11^**
	+	+	+	+	+	+	+	+	+	+	+	+	+	+
F17	**3.02 × 10^−11^**	**3.82 × 10^−10^**	**5.87 × 10^−04^**	**5.97 × 10^−09^**	7.51 × 10^00^	**1.69 × 10^−09^**	**1.46 × 10^−10^**	**3.34 × 10^−11^**	**1.20 × 10^−08^**	**2.37 × 10^−10^**	**1.78 × 10^−04^**	**2.84 × 10^−04^**	**1.39 × 10^−06^**	**4.94 × 10^−05^**
	+	+	+	+	=	+	+	+	+	+	+	+	+	+
F18	**3.02 × 10^−11^**	**3.02 × 10^−11^**	**3.02 × 10^−11^**	**3.02 × 10^−11^**	**3.02 × 10^−11^**	**3.02 × 10^−11^**	**3.02 × 10^−11^**	**3.02 × 10^−11^**	**3.02 × 10^−11^**	**3.02 × 10^−11^**	**3.02 × 10^−11^**	**3.02 × 10^−11^**	**3.02 × 10^−11^**	**3.02 × 10^−11^**
	+	+	+	+	+	+	+	+	+	+	+	+	+	+
F19	**3.02 × 10^−11^**	**5.87 × 10^−04^**	**3.02 × 10^−11^**	**3.02 × 10^−11^**	**3.02 × 10^−11^**	**3.02 × 10^−11^**	**3.02 × 10^−11^**	**3.02 × 10^−11^**	**3.02 × 10^−11^**	**3.02 × 10^−11^**	**3.02 × 10^−11^**	**3.02 × 10^−11^**	**3.02 × 10^−11^**	**3.02 × 10^−11^**
	+	+	+	+	+	+	+	+	+	+	+	+	+	+
F20	**3.02 × 10^−11^**	**3.02 × 10^−11^**	**3.02 × 10^−11^**	**3.02 × 10^−11^**	**3.02 × 10^−11^**	**3.02 × 10^−11^**	**3.02 × 10^−11^**	**3.02 × 10^−11^**	**3.02 × 10^−11^**	**3.02 × 10^−11^**	**3.02 × 10^−11^**	**3.02 × 10^−11^**	**3.02 × 10^−11^**	**3.02 × 10^−11^**
	+	+	+	+	+	+	+	+	+	+	+	+	+	+
F21	**6.72 × 10^−10^**	**3.99 × 10^−04^**	**7.66 × 10^−05^**	**2.15 × 10^−10^**	**9.06 × 10^−08^**	**3.02 × 10^−11^**	**3.02 × 10^−11^**	**5.57 × 10^−10^**	**3.02 × 10^−11^**	**8.99 × 10^−11^**	**9.92 × 10^−11^**	**1.96 × 10^−10^**	**7.12 × 10^−09^**	**1.07 × 10^−07^**
	+	+	+	+	+	+	+	+	+	+	+	+	+	+
F22	**3.02 × 10^−11^**	**3.02 × 10^−11^**	**3.02 × 10^−11^**	**3.02 × 10^−11^**	**3.02 × 10^−11^**	**3.02 × 10^−11^**	**3.02 × 10^−11^**	**3.02 × 10^−11^**	**3.02 × 10^−11^**	**3.02 × 10^−11^**	**3.02 × 10^−11^**	**3.02 × 10^−11^**	**3.02 × 10^−11^**	**3.02 × 10^−11^**
	+	+	+	+	+	+	+	+	+	+	+	+	+	+
F23	**3.02 × 10^−11^**	**3.02 × 10^−11^**	**3.02 × 10^−11^**	**3.02 × 10^−11^**	**3.02 × 10^−11^**	**3.02 × 10^−11^**	**3.02 × 10^−11^**	**3.02 × 10^−11^**	**3.02 × 10^−11^**	**3.02 × 10^−11^**	**3.02 × 10^−11^**	**3.02 × 10^−11^**	**3.02 × 10^−11^**	**3.02 × 10^−11^**
	+	+	+	+	+	+	+	+	+	+	+	+	+	+
F24	**3.02 × 10^−11^**	**3.02 × 10^−11^**	**3.02 × 10^−11^**	**3.02 × 10^−11^**	**3.02 × 10^−11^**	**3.02 × 10^−11^**	**3.02 × 10^−11^**	**3.02 × 10^−11^**	**3.02 × 10^−11^**	**3.02 × 10^−11^**	**3.02 × 10^−11^**	**3.02 × 10^−11^**	**3.02 × 10^−11^**	**3.02 × 10^−11^**
	+	+	+	+	+	+	+	+	+	+	+	+	+	+
F25	**3.02 × 10^−11^**	**3.34 × 10^−11^**	**3.02 × 10^−11^**	**3.02 × 10^−11^**	**3.02 × 10^−11^**	**3.02 × 10^−11^**	**3.02 × 10^−11^**	**3.02 × 10^−11^**	**3.02 × 10^−11^**	**3.02 × 10^−11^**	**3.02 × 10^−11^**	**3.02 × 10^−11^**	**3.02 × 10^−11^**	**3.02 × 10^−11^**
	+	+	+	+	+	+	+	+	+	+	+	+	+	+
F26	**3.02 × 10^−11^**	**2.43 × 10^−05^**	**1.39 × 10^−06^**	**3.02 × 10^−11^**	**9.92 × 10^−11^**	**3.02 × 10^−11^**	**3.02 × 10^−11^**	**3.02 × 10^−11^**	**3.02 × 10^−11^**	**3.02 × 10^−11^**	**5.49 × 10^−11^**	**3.34 × 10^−11^**	**3.02 × 10^−11^**	**6.07 × 10^−11^**
	+	+	+	+	+	+	+	+	+	+	+	+	+	+
F27	**3.02 × 10^−11^**	**3.02 × 10^−11^**	**3.02 × 10^−11^**	**3.02 × 10^−11^**	**3.02 × 10^−11^**	**3.02 × 10^−11^**	**3.02 × 10^−11^**	**3.02 × 10^−11^**	**3.02 × 10^−11^**	**3.02 × 10^−11^**	**3.02 × 10^−11^**	**3.02 × 10^−11^**	**3.02 × 10^−11^**	**3.02 × 10^−11^**
	+	+	+	+	+	+	+	+	+	+	+	+	+	+
F28	**3.02 × 10^−11^**	**3.02 × 10^−11^**	**3.02 × 10^−11^**	**3.02 × 10^−11^**	**3.02 × 10^−11^**	**3.02 × 10^−11^**	**3.02 × 10^−11^**	**3.02 × 10^−11^**	**3.02 × 10^−11^**	**3.02 × 10^−11^**	**3.02 × 10^−11^**	**3.02 × 10^−11^**	**3.02 × 10^−11^**	**3.02 × 10^−11^**
	+	+	+	+	+	+	+	+	+	+	+	+	+	+
F29	**3.02 × 10^−11^**	**3.02 × 10^−11^**	**3.02 × 10^−11^**	**3.02 × 10^−11^**	**3.02 × 10^−11^**	**3.02 × 10^−11^**	**3.02 × 10^−11^**	**3.02 × 10^−11^**	**3.02 × 10^−11^**	**3.02 × 10^−11^**	**3.02 × 10^−11^**	**3.02 × 10^−11^**	**3.02 × 10^−11^**	**3.02 × 10^−11^**
	+	+	+	+	+	+	+	+	+	+	+	+	+	+

**Table 5:** ImWOA’s sensitivity to the parameter changes.

Dim = 100				
Func.	Index	$w_{1}$:$w_{2}$ = 1:1	$w_{1}$:$w_{2}$ = 2:1	$w_{1}$:$w_{2}$ = 3:1
F1	Mean	4.18 × 10^5^	1.37 × 10^4^	7.88 × 10^6^
	rank	2	1	3
F2	Mean	9.02 × 10^5^	3.70 × 10^3^	6.12 × 10^4^
	rank	3	1	2
F3	Mean	3.33 × 10^4^	5.48 × 10^2^	1.25 × 10^3^
	rank	3	1	2
F4	Mean	3.24 × 10^3^	8.62 × 10^2^	5.14 × 10^4^
	rank	2	1	3
F5	Mean	2.13 × 10^2^	5.00 × 10^2^	9.45 × 10^3^
	rank	1	2	3
F6	Mean	3.22 × 10^4^	1.88 × 10^3^	5.64 × 10^5^
	rank	2	1	3
F7	Mean	3.25 × 10^4^	7.00 × 10^2^	9.52 × 10^3^
	rank	3	1	2
F8	Mean	2.56 × 10^3^	9.01 × 10^2^	7.33 × 10^4^
	rank	2	1	3
F9	Mean	3.25 × 10^5^	1.80 × 10^4^	9.22 × 10^7^
	rank	2	1	3
F10	Mean	6.66 × 10^6^	2.54 × 10^4^	1.22 × 10^3^
	rank	3	2	1
F11	Mean	3.56 × 10^7^	2.15 × 10^6^	4.98 × 10^8^
	rank	2	1	3
F12	Mean	3.45 × 10^6^	2.17 × 10^4^	6.78 × 10^5^
	rank	3	1	2
F13	Mean	4.65 × 10^7^	3.09 × 10^6^	7.42 × 10^8^
	rank	2	1	3
F14	Mean	6.82 × 10^7^	1.53 × 10^5^	2.81 × 10^6^
	rank	3	1	2
F15	Mean	2.56 × 10^5^	6.80 × 10^3^	8.61 × 10^4^
	rank	3	1	2
F16	Mean	2.56 × 10^4^	3.94 × 10^3^	7.42 × 10^5^
	rank	2	1	3
F17	Mean	1.25 × 10^7^	4.82 × 10^6^	6.42 × 10^8^
	rank	2	1	3
F18	Mean	9.42 × 10^5^	8.39 × 10^3^	2.16 × 10^4^
	rank	3	1	2
F19	Mean	1.23 × 10^3^	2.48 × 10^3^	3.78 × 10^5^
	rank	1	2	3
F20	Mean	5.32 × 10^4^	2.66 × 10^3^	6.48 × 10^5^
	rank	2	1	3
F21	Mean	4.25 × 10^5^	2.79 × 10^3^	6.94 × 10^4^
	rank	3	1	2
F22	Mean	8.53 × 10^5^	2.49 × 10^3^	5.32 × 10^4^
	rank	3	1	2
F23	Mean	6.32 × 10^4^	2.59 × 10^3^	6.31 × 10^5^
	rank	2	1	3
F24	Mean	2.68 × 10^2^	3.31 × 10^3^	7.42 × 10^4^
	rank	1	2	3
F25	Mean	9.72 × 10^4^	5.97 × 10^3^	9.75 × 10^2^
	rank	3	2	1
F26	Mean	6.32 × 10^4^	3.44 × 10^3^	5.31 × 10^5^
	rank	2	1	3
F27	Mean	5.32 × 10^4^	2.72 × 10^3^	8.72 × 10^5^
	rank	2	1	3
F28	Mean	5.33 × 10^4^	1.98 × 10^5^	8.62 × 10^6^
	rank	1	2	3
F29	Mean	9.96 × 10^6^	9.96 × 10^5^	9.96 × 10^4^
	rank	3	2	1
Average Rank		2.276	1.241	2.483
Combined Rank		2	1	3

**Table 6:** Comparative performance statistics of multi-run independent experiments.

Algorithms	ImWOA	WOA	E-WOA	IWOA	IWOSSA	RAV-WOA	WOAAD
Mean	147,041.24	241,639.96	249,941.32	239,785.64	237,602.55	167,897.84	251,044.01
rank	1	5	6	4	3	2	7

## Data Availability

The raw data supporting the conclusions of this article will be made available by the authors on request.

## References

[1] Zhang Y., Jin Z. (2022). Comprehensive learning Jaya algorithm for engineering design optimization problems. J. Intell. Manuf..

[2] Kalpana P., Nagendra Prabhu S., Polepally V., Rao D.B.J. (2022). Exponentially-spider monkey optimization based allocation of resource in cloud. Int. J. Intell. Syst..

[3] Nai C. (2023). Energy finance risk warning model based on GABP algorithm. Front. Energy Res..

[4] Rambabu D., Govardhan A. (2023). Optimization assisted frequent pattern mining for data replication in cloud: Combining sealion and grey wolf algorithm. Adv. Eng. Softw..

[5] Phan T., Sell D., Wang E.W., Doshay S., Edee K., Yang J., Fan J.A. (2019). High-efficiency, large-area, topology-optimized metasurfaces. Light Sci. Appl..

[6] Berger K., Rivera Caicedo J.P., Martino L., Wocher M., Hank T., Verrelst J. (2021). A survey of active learning for quantifying vegetation traits from terrestrial earth observation data. Remote Sens..

[7] Lee C.C., Hussain J., Chen Y. (2022). The optimal behavior of renewable energy resources and government’s energy consumption subsidy design from the perspective of green technology implementation. Renew. Energy.

[8] Zamir M., Abdeljawad T., Nadeem F., Wahid A., Yousef A. (2021). An optimal control analysis of a COVID-19 model. Alex. Eng. J..

[9] Wu L., Huang X., Cui J., Liu C., Xiao W. (2023). Modified adaptive ant colony optimization algorithm and its application for solving path planning of mobile robot. Expert Syst. Appl..

[10] Taher F., Abdel-Salam M., Elhoseny M., El-Hasnony I.M. (2023). Reliable machine learning model for IIoT botnet detection. IEEE Access.

[11] Llopis-Albert C., Rubio F., Zeng S. (2023). Multiobjective optimization framework for designing a vehicle suspension system. A comparison of optimization algorithms. Adv. Eng. Softw..

[12] Elhoseny M., Abdel-Salam M., El-Hasnony I.M. (2024). An improved multi-strategy Golden Jackal algorithm for real world engineering problems. Knowl.-Based Syst..

[13] Askr H., Abdel-Salam M., Hassanien A.E. (2024). Copula entropy-based golden jackal optimization algorithm for high-dimensional feature selection problems. Expert Syst. Appl..

[14] Abdel-Salam M., Kumar N., Mahajan S. (2024). A proposed framework for crop yield prediction using hybrid feature selection approach and optimized machine learning. Neural Comput. Appl..

[15] Abdel-Salam M., Hassanien A.E. (2024). A novel dynamic chaotic golden jackal optimization algorithm for sensor-based human activity recognition using smartphones for sustainable smart cities. Artificial Intelligence for Environmental Sustainability and Green Initiatives.

[16] Zhang J., Ning Z., Ali R.H., Waqas M., Tu S., Ahmad I. (2023). A many-objective ensemble optimization algorithm for the edge cloud resource scheduling problem. IEEE Trans. Mob. Comput..

[17] Sulaiman M.H., Mustaffa Z., Saari M.M., Daniyal H. (2020). Barnacles mating optimizer: A new bio-inspired algorithm for solving engineering optimization problems. Eng. Appl. Artif. Intell..

[18] Wang G.G., Deb S., Coelho L.D.S. (2018). Earthworm optimisation algorithm: A bio-inspired metaheuristic algorithm for global optimisation problems. Int. J. Bio-Inspired Comput..

[19] Dhiman G., Kumar V. (2019). Seagull optimization algorithm: Theory and its applications for large-scale industrial engineering problems. Knowl.-Based Syst..

[20] Kaur S., Awasthi L.K., Sangal A.L., Dhiman G. (2020). Tunicate Swarm Algorithm: A new bio-inspired based metaheuristic paradigm for global optimization. Eng. Appl. Artif. Intell..

[21] Shi Y. (2011). Brain storm optimization algorithm. Proceedings of the International Conference in Swarm Intelligence.

[22] Askari Q., Saeed M., Younas I. (2020). Heap-based optimizer inspired by corporate rank hierarchy for global optimization. Expert Syst. Appl..

[23] Dehghani M., Trojovský P. (2021). Teamwork optimization algorithm: A new optimization approach for function minimization/maximization. Sensors.

[24] Talatahari S., Azizi M. (2021). Chaos game optimization: A novel metaheuristic algorithm. Artif. Intell. Rev..

[25] Mirjalili S. (2016). SCA: A sine cosine algorithm for solving optimization problems. Knowl.-Based Syst..

[26] Abedinpourshotorban H., Shamsuddin S.M., Beheshti Z., Jawawi D.N. (2016). Electromagnetic field optimization: A physics-inspired metaheuristic optimization algorithm. Swarm Evol. Comput..

[27] Karaboga D. (2010). Artificial bee colony algorithm. Scholarpedia.

[28] Yang X.S., Deb S. (2009). Cuckoo search via Lévy flights. Proceedings of the 2009 World Congress on Nature & Biologically Inspired Computing.

[29] Wang G.G., Deb S., Coelho L.S. (2015). Elephant herding optimization. Proceedings of the 2015 3rd International Symposium on Computational and Business Intelligence.

[30] Masadeh R., Mahafzah B.A., Sharieh A. (2019). Sea lion optimization algorithm. Int. J. Adv. Comput. Sci. Appl..

[31] Mirjalili S., Lewis A. (2016). The whale optimization algorithm. Adv. Eng. Softw..

[32] Liang Z., Shu T., Ding Z. (2024). A novel improved whale optimization algorithm for global optimization and engineering applications. Mathematics.

[33] Liu L., Zhang R. (2022). Multistrategy improved whale optimization algorithm and its application. Comput. Intell. Neurosci..

[34] Chakraborty S., Sharma S., Saha A.K., Saha A. (2022). A novel improved whale optimization algorithm to solve numerical optimization and real-world applications. Artif. Intell. Rev..

[35] Sun G., Shang Y., Zhang R. (2022). An efficient and robust improved whale optimization algorithm for large scale global optimization problems. Electronics.

[36] Shen Y., Zhang C., Gharehchopogh F.S., Mirjalili S. (2023). An improved whale optimization algorithm based on multi-population evolution for global optimization and engineering design problems. Expert Syst. Appl..

[37] Li M., Yu X., Fu B., Wang X. (2023). A modified whale optimization algorithm with multi-strategy mechanism for global optimization problems. Neural Comput. Appl..

[38] Wu G., Mallipeddi R., Suganthan P.N. (2017). Problem Definitions and Evaluation Criteria for the CEC 2017 Competition on Constrained Real-Parameter Optimization.

[39] Kennedy J., Eberhart R. (1995). Particle swarm optimization. Proceedings of the ICNN’95-International Conference on Neural Networks.

[40] Simon D. (2008). Biogeography-based optimization. IEEE Trans. Evol. Comput..

[41] Li S., Chen H., Wang M., Heidari A.A., Mirjalili S. (2020). Slime mould algorithm: A new method for stochastic optimization. Future Gener. Comput. Syst..

[42] Storn R., Price K. (1997). Differential evolution—A simple and efficient heuristic for global optimization over continuous spaces. J. Glob. Optim..

[43] Mirjalili S., Mirjalili S.M., Lewis A. (2014). Grey wolf optimizer. Adv. Eng. Softw..

[44] Xue J., Shen B. (2020). A novel swarm intelligence optimization approach: Sparrow search algorithm. Syst. Sci. Control Eng..

[45] Heidari A.A., Mirjalili S., Faris H., Aljarah I., Mafarja M., Chen H. (2019). Harris hawks optimization: Algorithm and applications. Future Gener. Comput. Syst..

[46] Karaboga D. (2005). An Idea Based on Honey Bee Swarm for Numerical Optimization.

[47] Nadimi-Shahraki M.H., Zamani H., Mirjalili S. (2022). Enhanced whale optimization algorithm for medical feature selection: A COVID-19 case study. Comput. Biol. Med..

[48] Xiong G., Zhang J., Shi D., He Y. (2018). Parameter extraction of solar photovoltaic models using an improved whale optimization algorithm. Energy Convers. Manag..

[49] Saafan M.M., El-Gendy E.M. (2021). IWOSSA: An improved whale optimization salp swarm algorithm for solving optimization problems. Expert Syst. Appl..

[50] Ma G., Yue X. (2022). An improved whale optimization algorithm based on multilevel threshold image segmentation using the Otsu method. Eng. Appl. Artif. Intell..

[51] Tang J., Wang L. (2024). A whale optimization algorithm based on atom-like structure differential evolution for solving engineering design problems. Sci. Rep..

